# Molecular insights into ligand recognition and G protein coupling of the neuromodulatory orphan receptor GPR139

**DOI:** 10.1038/s41422-021-00591-w

**Published:** 2021-12-17

**Authors:** Yali Zhou, Henrik Daver, Boris Trapkov, Lijie Wu, Meng Wu, Kasper Harpsøe, Patrick R. Gentry, Kaiwen Liu, Marina Larionova, Junlin Liu, Na Chen, Hans Bräuner-Osborne, David E. Gloriam, Tian Hua, Zhi-Jie Liu

**Affiliations:** 1 iHuman Institute, ShanghaiTech University, Shanghai, China; 2 School of Life Science and Technology, ShanghaiTech University, Shanghai, China; 3 University of Chinese Academy of Sciences, Beijing, China; 4 CAS Center for Excellence in Molecular Cell Science, Shanghai Institute of Biochemistry and Cell Biology, Chinese Academy of Sciences, Shanghai, China; 5 Department of Drug Design and Pharmacology, University of Copenhagen, Universitetsparken 2, Copenhagen, Denmark; 6 Photobiology laboratory, Institute of Biophysics SB RAS, Federal Research Center “Krasnoyarsk Science Center SB RAS”, Akademgorodok 50/50, Krasnoyarsk, Russia

**Keywords:** Cryoelectron microscopy, Extracellular signalling molecules

Dear Editor,

The G protein-coupled receptor GPR139 is involved in neuromodulation, and one of its agonists is in clinical trials for the treatment of cognitive impairment and negative symptoms of schizophrenia. While GPR139 is a understudied ‘orphan’ receptor, it can be activated by the amino acids l-Trp, l-Phe^1^ or α-Melanocyte-stimulating hormone (α-MSH) which is an endogenous agonist of melanocortin receptors.^2,3^ GPR139 activation triggers several G protein pathways of which G_q/11_ is the primary.^4–7^ Of note, the expression of GPR139 correlates with that of the μ-opioid and dopamine D_2_ receptors in a broad range of the central nervous system (CNS), which acts as a regulator of μ-opioid and dopamine signaling.^6–9^ For example, GPR139 antagonist JNJ-3792165 increases the sensitivity of the μ-opioid receptor to morphine.^6^ Hitherto, the structural basis of how ligands interact with and activate GPR139 to transduce diverse signals has remained unknown. Furthermore, the more physiologically relevant intermediate states of GPCR–G protein complex structures in the nucleotide-bound forms are also elusive. Here, we report the cryo-electron microscopy (cryo-EM) structures of GPR139 in complex with a key reference ligand JNJ-63533054 which is an analog of agent TAK-041^10^ in clinical trial, and miniG_s/q_ or G_i_ in nucleotide-free form, as well as the GPR139–JNJ-63533054–miniG_s/q_ complex in GDP- and GTP-bound states, respectively (Fig. 1a–f).

**Fig. 1:**
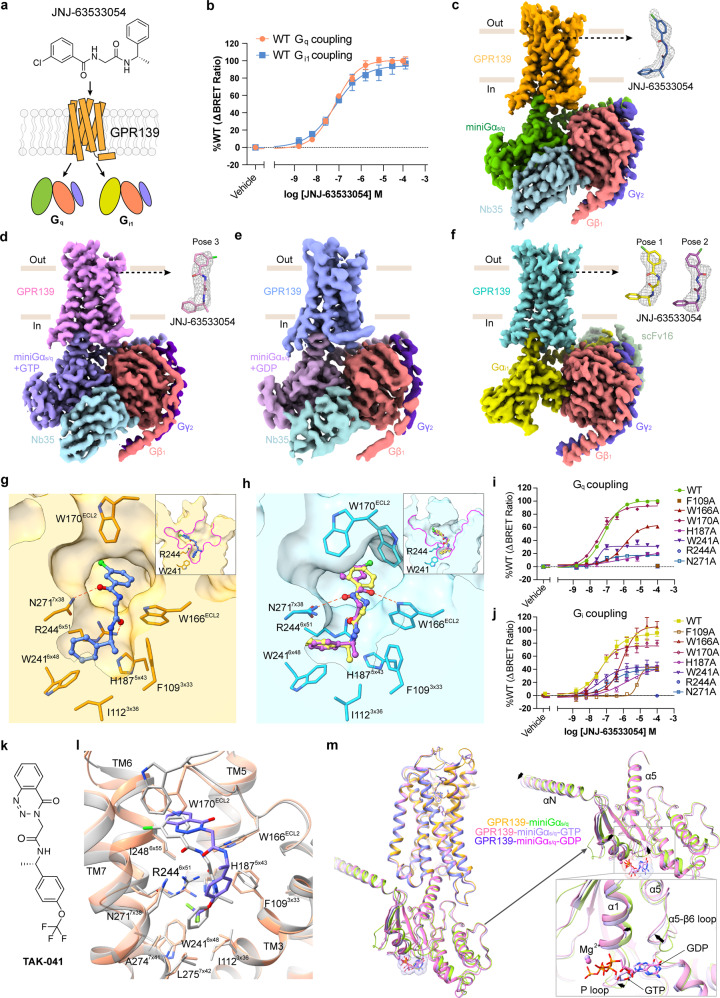
Cryo-EM structures of JNJ-63533054-bound GPR139–G protein complexes in different states, and analyses of the binding between surrogate ligands JNJ-63533054/TAK-041 and GPR139. **a**, **b** GPR139 agonist JNJ-63533054 induces G_q_ and G_i_ protein coupling in the BRET assay. **c** Cryo-EM density map of the GPR139–JNJ-63533054–miniG_s/q_–Nb35 complex in nucleotide-free state with density map for JNJ-63533054. **d** Cryo-EM density map of GTP-bound GPR139–JNJ-63533054–miniG_s/q_–Nb35 complex with density map for JNJ-63533054 (pose-3). **e** Cryo-EM density map of GDP-bound GPR139–JNJ-63533054–miniG_s/q_–Nb35 complex. **f** Cryo-EM density map of GPR139–JNJ-63533054–G_i_–scFv16 complex in nucleotide-free state with density maps for JNJ-63533054. Based on the density map, JNJ-63533054 adopts two poses (pose-1 and pose-2). **g** Key residues (orange sticks) involved in JNJ-63533054 (blue sticks) binding in the GPR139–miniG_s/q_ complex. **h** Key residues (cyan sticks) involved in JNJ-63533054 (pose-1, yellow sticks; pose-2, magenta sticks) binding in the GPR139–G_i_ complex. Note that W170^ECL2^ adapts to the two conformations. **i**, **j** BRET assay validation of key residues involved in ligand binding. **k**, **l** Chemical structure of the GPR139 agonist TAK-041 (Takada, **k**) and its docking pose in GPR139 (**l**). **m** The overall structure comparison of GTP-bound, GDP-bound and nucleotide-free miniGα_s/q_–GPR139 complexes. GTP- and GDP-binding areas are zoomed in.

To improve the expression and homogeneity of GPR139, a Xylanase fusion protein was fused at the N-terminus; the C-terminal residues 322–353 were truncated, and a S62^2×39^V mutation was also introduced (superscript denotes generic residue numbers^11^). The S62^2×39^V mutation has no effect on the potency of surrogate agonist JNJ-63533054 (Supplementary information, Table S1). The stable GPR139–G_q_ complex consisting of purified GPR139, miniG_s/q_ protein^12^ and Nb35 in the presence of agonist JNJ-63533054 was obtained in vitro. Importantly, three cryo-EM structures of JNJ-63533054-bound GPR139–miniG_s/q_ complex in the nucleotide-free (hereafter referred to as GPR139–miniG_s/q_), GDP-bound and GTP-bound states were determined at global resolutions of 3.2 Å, 3.3 Å and 3.8 Å, respectively (Fig. 1c–e; Supplementary information, Figs. S1–S3 and Table S5). To further investigate the structural basis of G protein selectivity of GPR139 (Fig. 1a, b), we also determined the structure of JNJ-63533054-bound GPR139–G_i_ at 3.2 Å resolution, by co-expression of the receptor with the heterotrimeric G_i1_ protein and stabilizing scFv16^13^ (Fig. 1f; Supplementary information, Fig. S4 and Table S5). The overall structures of the receptors in the two complexes are quite similar, with a root mean square deviation (RMSD) value for Cα atoms of 1.47 Å. The main differences are found in extracellular loops 2 and 3 (ECL2 and ECL3), and the intracellular regions of TM5 and TM6 that bind the G proteins (Supplementary information, Figs. S5, S6).

The agonist JNJ-63533054 binds in the prototypical ligand pocket in the bundle of seven transmembrane helices in both the GPR139–miniG_s/q_ and GPR139–G_i_ structures. In the GPR139–miniG_s/q_ structure, the chlorophenyl moiety of JNJ-63533054 is situated in a hydrophobic pocket lined by the residues I169^ECL2^, W170^ECL2^, M247^6×54^, I248^6×55^, H251^6×58^ and V263^7×30^, and its chloride points toward the gap between TM6 and TM7 (Supplementary information, Fig. S7a). The carbonyl oxygen, O1, in JNJ-63533054 forms a weak hydrogen bond with the ND2 of residue N271^7×38^. The carbonyl oxygen O2 from JNJ-63533054 establishes a hydrogen bond with the NH2 of residue R244^6×51^. The JNJ-63533054’s phenyl ring is buried in a deep hydrophobic pocket formed by I80^2×57^, F85^2×62^, F109^3×33^, I112^3×36^ and L275^7×42^ (Fig. 1g; Supplementary information, Fig. S7a, f), where it has cation–π interaction with residue R244^6×51^and π–π interaction with W241^6×48^. Our mutagenesis analyses provide further evidence for the roles of F109^3×33^, H187^5×43^ and N271^7×38^ in GPR139 activation (Fig. 1i; Supplementary information, Fig. S7d, e and Table S2).

Interestingly, in the GPR139–G_i_ structure, two binding poses of JNJ-63533054 are identified based on the EM map (Fig. 1f). ‘Pose-1’ is similar to that in the GPR139–miniG_s/q_ structure, while ‘Pose-2’ has a flipped amide bond linking the chlorophenyl ring and N1 of JNJ-63533054, and is associated with a changed direction of the chloride and carbonyl oxygen (Fig. 1h; Supplementary information, Fig. S7b). Concordantly, our MD simulations show similar flexibility of the JNJ-63533054 binding mode (Supplementary information, Fig. S8a, b). For pose-2, the ligand’s N2 forms a hydrogen bond to E108^3×32^. In addition, W170^ECL2^ adopts two conformations, whereof ‘conf-1’ is similar to that in the GPR139–miniG_s/q_ structure and in ‘conf-2’ the sidechain swings away from the ligand-binding pocket (Supplementary information, Fig. S8c, d). Interestingly, the MD simulations show the correlation between the binding poses of JNJ-63533054 with the conformations of W170^ECL2^ (Supplementary information, Fig. S8e–g). In addition, the interaction between W166^ECL2^ and JNJ-63533054 is dependent on the ligand conformation as well. In the case of pose-1, JNJ-63533054’s N2 forms a hydrogen bond with residue N271^7×38^ and N1 has weak repulsion with NE1 of residue W166^ECL2^. For pose-2, carbonyl oxygen O1 forms a hydrogen bond with NE1 of residue W166^ECL2^ (Fig. 1h; Supplementary information, Fig. S7b). Furthermore, the W166^ECL2^A mutation results in a one order of magnitude potency decrease in both G_q_ and G_i_ BRET assays (Fig. 1i, j; Supplementary information, Fig. S7d, e and Table S2).

Unexpectedly, in the structure of GTP-bound GPR139–miniG_s/q_ complex, JNJ-63533054 adopts another binding pose ‘Pose-3’ that is almost identical to the binding pose of JNJ-63533054 in the nucleotide-free GPR139–miniG_s/q_ structure, except that there is a 180-degree flip on the (O=)C–C (chlorophenyl) bond (Fig. 1d; Supplementary information, Fig. S7c). This binding pose change is apparently due to the more converged ECL2 with better density map in the GTP-bound GPR139–miniG_s/q_ complex structure. In GDP-bound GPR139–miniG_s/q_ structure, JNJ-63533054 adopts a similar binding pose as in the nucleotide-free GPR139–miniG_s/q_ structure. Therefore, the different binding poses of JNJ-63533054 in the nucleotide-free miniG_s/q_ and G_i_, as well as nucleotide-bound miniG_s/q_ complex structures are correlated to the coupling of different G protein forms. To fully understand these results, further investigation, especially solving more nucleotide-bound GPCR–G protein complex structures, is needed.

To decipher the structural basis of the activation of GPR139, we first investigated R244^6*×*51^ that points into the ligand-binding pocket and forms charged interactions with JNJ-63533054 (Fig. 1g, h), which may be important for GPR139 activation. Of note, this arginine, R244, only appears at position 6×51 in GPR139 and its close homolog GPR142. Other class A GPCRs instead have other amino acids in this position whereof Y and F are most conserved (Supplementary information, Fig. S9a). When mutating R244^6×51^ to different residues, we found that the K mutant has lower potency for JNJ-63533054, and that all other mutants almost abolish the expression and/or activity (Supplementary information, Fig. S9b).^14^ These results show that a positively charged residue at position 6×51 is critical for ligand recognition and activation of GPR139. Interestingly, JNJ-63533054 binds deeper in the GPR139–miniG_s/q_ structure and is closer to the toggle-switch residue W241^6*×*48^ compared with that of other solved active GPCR structures (Supplementary information, Fig. S9c). Finally, GPR139 shares a hallmark of active-state class A GPCR structures, an ‘unwinding’ of TM7 in the cytosolic part, which is stabilized by a microswitch residue, Y^7×53^, in the conserved NPxxY^7×49–53^ motif. In 94% of class A GPCRs, such a conformational change in TM7 comes from a helix kink enabled by the P^7×50^ residue (Supplementary information, Fig. S9d, e). Notably, GPR139 instead contains an F at this position. We found that mutation to the canonical P residue almost abolishes G_q_ and G_i_ coupling — demonstrating an important role of F^7×50^ in signal transduction (Supplementary information, Table S1). The sidechain of F^7×50^ protrudes into a predominantly hydrophobic core formed by residues from the cytoplastic end of TM1, F287^7×55^ and F292^8×50^, and this interaction may facilitate TM7’s conformational change that favors receptor activation (Supplementary information, Fig. S9f).

Since GPR139 is a therapeutic target for several diseases, we performed molecular docking of TAK-041, which is in clinical trial by Takeda, and Cmp1a from H. Lundbeck A/S. The top-ranked docking poses resemble the binding mode of JNJ-63533054 in GPR139 (Fig. 1k, l; Supplementary information, Fig. S10a, b). They form similar interactions with the residues observed in the JNJ-63533054-binding pocket. Concordantly, our pharmacological analysis on the key residues greatly attenuates the potency and efficacy of TAK-041 and Cmp1a (Supplementary information, Fig. S10c–f and Table S2). Interestingly, mutations W166^ECL2^A and W241^6×48^A have major effects on Cmp1a and JNJ-63533054, but only minor to no effect on TAK-041 (Supplementary information, Table S2), which is consistent with TAK-041 forming weak interactions with the two residues. Together, these results confirm that the three agonists JNJ-63533054, TAK-041 and Cmp1a developed by different pharmaceutical companies largely share binding sites, but also have distinct differences in activating GPR139.

Furthermore, we investigated the binding modes of the endogenous amino acids l-Trp and l-Phe in GPR139. The docking results showed that l-Trp and l-Phe share binding site in the buried pocket of JNJ-63533054. Both the sidechain and the carboxyl group of l-Trp or l-Phe interact with R244^6×51^, while their amine functionalities are positioned towards residues D84^2×61^, E105^3×29^ and E108^3×32^ in TM2–3 of the receptor (Supplementary information, Fig. S10g, h). Finally, to pinpoint residues that may offer ligand selectivity for GPR139 over its close homolog GPR142, which is involved in the regulation of glucose metabolism and is a proposed target for the treatment of type 2 diabetes,^15^ we generated single-point mutations of residues that are located in the ligand-binding pocket and differ between GPR139 and GPR142 (Supplementary information, Fig. S10i). We found that only the I112^3×36^N (I in GPR139, N in GPR142) mutation abrogates the activity of all three tested surrogate ligands in GPR139 — likely by hindering binding of the buried part (Supplementary information, Fig. S10j and Tables S3, S4). These results will help to explain observed ligand selectivity and provide clues for the rational design of more selective ligands targeting GPR139 or GPR142. In addition, we found that a wall of seven negatively charged receptor residues on TM2, TM3 and TM7 spanning all the way from the extracellular face to the bottom of the transmembrane helix cavity (Supplementary information, Fig. S11). Of these, D84^2*×*61^, E105^3×29^ and E108^3×32^ are located in the ligand-binding pocket but have no interactions with JNJ-63533054. However, the D84^2×61^A, D89^2×66^A and D268^7×35^A mutations affect either the efficacy or potency of JNJ-63533054 (Supplementary information, Table S4), indicating that the negatively charged wall may play a role in the entrance of the ligand to the binding pocket. However, these negatively charged residues are not conserved in GPR142, and their function needs to be further explored.

The structures of GDP- or GTP-bound intermediate states of the GPCR–G protein complex are very important for understanding the molecular mechanism of the dynamic process of GPCR–G protein interaction and signaling in more physiologically relevant conditions. Here, we determined the structures of nucleotide-bound GPR139–JNJ-63533054–miniG_s/q_ using optimized procedures on adding GDP or GTP prior to the cryo-EM sample freezing. In order to inhibit GTP-mediated dissociation of the receptor–G protein complex, an I372A mutation was introduced to the α5 of miniG_s/q_.^16^ The GDP and GTP are well-defined in EM density maps and the overall structure is similar to that of GPR139–miniG_s/q_ in the nucleotide-free state, except for the binding pose change of JNJ-63533054 and more compact ECL2 in the GTP-bound GPR139–miniG_s/q_ structure discussed above. Regarding the Gα subunits in the GDP-, GTP-bound and nucleotide-free states, the nucleotide binding induces noticeable conformation shifts of the β6–α5 loop and the P-loop, which connects the β1 strand and the α1 helix, to accommodate GTP or GDP binding (Fig. 1m).

Compared to the crystal structure of GDP-bound Gα_s_ (PDB: 6EG8), the Gα_s/q_ in the GDP-bound GPR139–miniG_s/q_ structure shows significant conformational changes, especially the α5 helix of Gα_s/q_. The bent α5 in the GDP-bound Gα_s_ crystal structure becomes straight, shifts one helix turn up and screws into the receptor core in the GDP-bound GPR139–miniG_s/q_ structure (Supplementary information, Fig. S12a). Concordantly, the last α5 helix turn is unwound to accommodate its upward movement. Furthermore, when comparing the crystal structure of GTP-bound Gα_s_ (PDB: 1AZT) with GTP-bound Gα_s/q_ in the GPR139–miniG_s/q_ structure, almost identical conformational changes in the α5 helix are observed (Supplementary information, Fig. S12b). In addition, the αN helix of Gα in GTP-bound GPR139–miniG_s/q_ structure is observed and forms weak interactions with Gβγ, whereas αN is disordered in the GTP-bound Gα crystal structure due to the absence of receptor and Gβγ.

In this study, structures of four different forms of the GPR139 complex provide mechanistic insights into the activation and signaling of GPR139. The interaction between the special R244^6×51^ and agonist JNJ-63533054 declares the unconventional theme of ligand selectivity and receptor activation in GPR139. When comparing the interactions between R244^6×51^ with three surrogate agonists (Fig. 1), we observed that the dumbbell-shaped agonists have polar peptide-like handles which form hydrogen bond with R244^6×51^. The bottom end of the dumbbell has a hydrophobic moiety, which is in the proximity of W241^6×48^, while its top end is more polar and extends towards the extracellular direction. We thus speculate that the above-mentioned characteristics of the dumbbell-shaped compounds are the key elements for ligand selectivity, which may guide future drug design for GPR139. Moreover, chronic pain and prescription opioid abuse are extremely prevalent worldwide and opioid misuse can be life-threatening. This study should facilitate the discovery of novel therapeutics to significantly increase the safety window of opioid-based pain reliever drugs.

Additionally, JNJ-63533054 exhibits different binding poses which are correlated to the coupling of different G protein subtypes or states, such as G_s/__q_, G_i,_ GTP- or GDP-bound G_s/__q_. Those observations provide evidence for G proteins’ allosteric modulation roles in GPCRs. The successful capture of GDP- or GTP-bound GPR139–miniG_s/q_ complex structures disclose the existence of two intermediate states during the G protein activation, which are the snapshots at the stage of GDP-bound miniG_s/q_ coupling to GPR139 and prior to the disassociation of GTP-bound miniG_s/q_ from GPR139.

## Supplementary information

Supplementary Material

## Data Availability

Coordinates and structure factors have been deposited in the Protein Data Bank for GPR139–JNJ-63533054–G_i_ (PDB: 7VUG, EMDB: EMD-32127), GPR139–JNJ-63533054–miniG_s/q_ (PDB: 7VUH, EMDB: EMD-32128), GTP-bound GPR139–JNJ-63533054–miniG_s/q_ (PDB: 7VUI, EMDB: EMD-32129) and GDP-bound GPR139–JNJ-63533054–miniG_s/q_ (PDB: 7VUJ, EMDB: EMD-32130).
