# Supplementary material for: Molecular insights into ligand recognition and G protein coupling of the neuromodulatory orphan receptor GPR139
Source: Cell Res. 2021 Dec 17;32(2):10. doi: 10.1038/s41422-021-00591-w (PMC8807744; doi:10.1038/s41422-021-00591-w)
Supplement: Supplementary file 1 — Supplementary Material [file 41422_2021_591_MOESM1_ESM.pdf]

Supplementary information

Methods

GPR139 construct design, expression and purification

The wild type (WT) GPR139 construct was cloned into the pFastBac1 vector with the N-terminal haemagglutinin signal peptide (HA) followed by a 10×His tag, Flag tag and a TEV protease site. To increase protein yield and homogeneity, a Xylanase (PDB: 2B45, MW 19.1 kDa) fusion protein was fused at the N-terminus of GPR139 and the C-terminal residues 322-353 were truncated. In addition, several mutations were designed based on a predicted GPR139 model and screened for stability and signaling activity. Eventually, S622x39V was selected and incorporated into the final GPR139-Xylanase expression construct.

Using the Bac-to-Bac baculovirus expression system (Invitrogen), the GPR139-Xylanase was expressed in *Spodoptera frugiperda* Sf9 insect cells, which were cultured at 27 °C and collected 48 h after infection. The cell pellets were lysed by washing and centrifugation using hypotonic buffer (10 mM HEPES pH 7.5, 20 mM KCl, 10 mM $MgCl_2$, and EDTA-free protease inhibitor cocktail (Roche) tablets), followed by high osmotic buffer (10 mM HEPES pH 7.5, 1 M NaCl, 20 mM KCl, 10 mM $MgCl_2$, and EDTA-free protease inhibitor cocktail). The purified membranes were resuspended with buffer (10 mM HEPES pH 7.5, 20 mM KCl, 10 mM $MgCl_2$, EDTA-free protease inhibitor cocktail, and 30% (v/v) glycerol) and stored at -80 °C for future use.

The purified membranes were incubated with 50 μM JNJ-63533054 for 2 h at 4 °C. Then, 1mg/mL iodoacetamide was added for another 1 h followed by the solubilization of GPR139-Xylanase in buffer containing 50 mM HEPES pH 7.5, 500 mM NaCl, 1% (w/v) lauryl maltose neopentyl glycol (LMNG, Anatrace), and 0.2% (w/v) cholesterol hemisucinate (CHS, Sigma-Aldrich) at 4 °C for 2.5–3 h. The supernatant was isolated by ultracentrifugation for 30 min, followed by incubation with TALON IMAC resin (Clontech) and 20 mM imidazole at 4 °C overnight. The resin was washed using 15 column volumes (CV) of wash buffer I (25 mM HEPES pH 7.5, 100 mM NaCl, 10% (v/v) glycerol, 0.05%(w/v) LMNG, 0.01% (w/v) CHS, 20 mM imidazole, and 25 μM JNJ-63533054), and 15 CV of wash buffer II (25 mM HEPES pH 7.5, 100 mM NaCl, 10% (v/v) glycerol, 0.01% (w/v) LMNG, 0.002% (w/v) CHS, 30 mM imidazole, and 25 μM JNJ-63533054). The resin was then eluted with 3 CV of elute buffer (25 mM HEPES pH 7.5, 100 mM NaCl, 10% (v/v) glycerol, 0.01% (w/v) LMNG, 0.002%(w/v) CHS, 250 mM imidazole, and 50 μM JNJ-63533054). The imidazole was removed by PD MiniTrap G-25 column (GE Health Care). The purified receptor was used for the following GPR139-G_q complex formation.

MiniG$\alpha_{s/q}$ construct design, expression and purification

The miniG$\alpha_{s/q}$, based on miniG$_{s/q}$70[1], was designed for the binding of Nb35 where the 6-25 amino acids of Gα_s were fused at the N-terminus, and was cloned into the pET-14a vector. MiniG$\alpha_{s/q}$ was expressed in the *E. coli* strain BL21 and induced at 20 °C for 20 h. Collected cell pellets were resuspended in buffer A (40 mM HEPES pH 7.5, 100 mM NaCl, 5 mM imidazole, 50 μM GDP, and 5 mM $MgCl_2$) with 100 μM DTT and EDTA-free complete protease inhibitor cocktail tablets, followed by sonication. The supernatant was collected after centrifugation and incubated with Ni-NTA resin at 4 °C for 2 h. The resin was then washed with 25 CV buffer B (20 mM HEPES pH 7.5, 250 mM NaCl, 10 mM imidazole, 50 μM GDP, 5 mM $MgCl_2$, and 10% Glycerol) and 10 CV buffer C (20 mM HEPES pH 7.5, 250 mM NaCl, 30 mM imidazole, 50 μM GDP, 1 mM $MgCl_2$, and 10% Glycerol). The target protein was eluted by 5 CV buffer D (20 mM HEPES pH 7.5, 50 mM NaCl, 500 mM imidazole, 50 μM GDP, 1 mM $MgCl_2$, and 10% Glycerol). The elution protein was digested by TEV protease and dialyzed in buffer E (20 mM HEPES pH 7.5, 50 mM NaCl, 10 μM GDP, 1 mM $MgCl_2$, and 10% Glycerol) overnight. Then the protein mixture was incubated with Ni-NTA resin to remove His tag and TEV protease. The digested protein was further purified by size-exclusion chromatography using a Superdex 75 column (GE healthcare). The purified miniG$\alpha_{s/q}$ protein was collected and concentrated to 4 mg/ml and stored at -80 °C for future complex building. The I372A mutated miniG$\alpha_{s/q}$ was purified by the same protocol.

G$\beta_1\gamma_2$ expression and purification

G$\beta_1\gamma_2$ subunits were cloned into the pFastBac Dual vector and expressed in Sf9 cell lines, which were cultured at 27 °C and collected 48 h after infection. The cell pellets were resuspended in buffer A (40 mM HEPES pH 7.5, 100 mM NaCl, 5 mM imidazole, and 5 mM $MgCl_2$) with 100 μM DTT and EDTA-free complete protease inhibitor cocktail tablets, and then were lysed by sonication. The supernatant was collected after centrifugation at 40,000 rpm for 30 min followed by incubation with Ni-NTA resin for 2 h. The resin was then washed with 15 CV buffer B (20 mM HEPES pH 7.5, 250 mM NaCl, 10 mM imidazole, and 10% Glycerol) and 10 CV buffer C (20 mM HEPES pH 7.5, 250 mM NaCl, 30 mM imidazole, and 10% Glycerol). The target protein was eluted by 5 CV buffer D (20 mM HEPES pH 7.5, 50 mM NaCl, 500 mM imidazole, and 10% Glycerol). The resulting elution was concentrated to 1 ml and diluted 10-fold with buffer E (20 mM HEPES pH 7.5, 50 mM NaCl, 1 mM DTT, and 10% Glycerol) to remove the imidazole before being loaded onto a 5 mL Hightrap Q HP column at a liner gradient of 50-500 mM NaCl. The protein peak for G$\beta_1\gamma_2$ was collected and concentrated to 3 mg/mL for forming miniG$_{s/q}$ heterotrimer.

GPR139-JNJ-63533054-miniG$_{s/q}$-Nb35 complex formation and purification

The purified GPR139 was mixed with a 1.5-fold molar excess of miniG$\alpha_{s/q}$, $\beta_1\gamma_2$ and Nb35. Nb35 was purified as previous reported [2]. The mixture was incubated at 24 °C for 1 h before adding 5 μl apyrase (0.5 U/μl) and incubated at 25 °C for 1 h. The mixture sample was then incubated at 4 °C overnight. The sample was loaded on to a Superdex 200 10/300 column in the equilibration buffer (20 mM HEPES, pH 7.5, 100 mM NaCl,

0.00075% (w/v) LMNG, 0.00025% (w/v) GDN, 0.0001% (w/v) CHS, 10 μM JNJ-63533054, and 100 mM TCEP). Peak fractions containing the GPR139-miniG$_{s/q}$-$\beta_1\gamma_2$-Nb35 complex were pooled and concentrated to 4 mg/ml for cryo-EM sample preparation.

GPR139-JNJ-63533054-miniG$_{s/q}$-Nb35-GDP/GTP complex formation and purification

For GPR139-JNJ-63533054-miniG$_{s/q}$-Nb35-GDP complex, the sample was prepared as nucleotide free samples. 1mM GDP and 2mM $MgCl_2$ were added 3 min before sample freezing. For GTP bound sample, the purified GPR139 was mixed with a 1.5-fold molar excess of miniG$\alpha_{s/q}$(I372A), $\beta_1\gamma_2$ and Nb35. Other steps were the same as before. 1mM GTP and 2mM $MgCl_2$ were added 3 min before sample freezing.

GPR139-JNJ-63533054-G$_i$-scFv16 complex formation and purification

The GPR139 and G$_i$ heterotrimer were co-expressed in Sf9 insect cells using the Bac-to-Bac Baculovirus Expression System (Invitrogen). Sf9 cells were infected at a cell density of 2-2.5×10^6 cells per mL with three separate virus preparations for GPR139, Gα_{i1} and G$\beta_1\gamma_2$ at a ratio of 2:1:1. The infected cells were cultured at 27 °C for 48 h before collection by centrifugation and the cell pellets were stored at -80 °C for future use.

The cell pellets corresponding to 2L GPR139-G$_i$ co-expression culture were thawed and lysed in the hypotonic buffer (50 mM HEPES, pH7.5, 2 mM $MgCl_2$, 50 mM NaCl, 50 μM JNJ-63533054, 4 μg/mL scFv16, 25 mU/mL apyrase, and EDTA-free complete protease inhibitor cocktail tablets (Roche)). scFv16 was purified as previously reported [3]. The lysate was incubated at room temperature for 2 h and then the membranes were collected by ultracentrifugation at 40,000 rpm for 30 min. The complex in membranes was solubilized in the solubilization buffer (50 mM HEPES pH 7.5, 500 mM NaCl, 1%(w/v) lauryl maltose neopentyl glycol (LMNG, Anatrace), 0.2% (w/v) cholesterol hemisucinate (CHS, Sigma-Aldrich), and 40 μM JNJ-63533054) at 4 °C for 2.5-3 h. The supernatant was isolated by ultracentrifugation and then incubated overnight with TALON IMAC resin (Clontech) and 20 mM imidazole at 4 °C. The resin was washed with 15 CV of washing buffer I (25 mM HEPES (pH 7.5), 100 mM NaCl, 10% (v/v) glycerol, 0.05% (w/v) LMNG, 0.01% (w/v) CHS, 20 mM imidazole, and 25 μM JNJ-63533054) and 15 CV of washing buffer II (25 mM HEPES (pH 7.5), 100 mM NaCl, 10% (v/v) glycerol, 0.01% (w/v) LMNG, 0.002% (w/v) CHS, 30 mM imidazole, and 25 μM JNJ-63533054). The protein was eluted using 3 CV of elution buffer (25mM HEPES pH 7.5, 100 mM NaCl, 10% (v/v) glycerol, 0.01% (w/v) LMNG, 0.002% (w/v) CHS, 250 mM imidazole, and 50 μM JNJ-63533054). The purified GPR139-G$_i$ complex was concentrated, then injected onto a Superdex200 10/300 GL column (GE Healthcare) in equilibration buffer (20 mM HEPES, pH 7.5, 100 mM NaCl, 0.00075% (w/v) LMNG, 0.00025%(w/v) GDN, 0.0001% (w/v) CHS, 10 μM JNJ-63533054, and 100 mM TCEP). The GPR139-JNJ-63533054-G$_i$-scFv16 complex peak fractions were collected and concentrated individually to 1.0-2.0 mg/mL for cryo-EM sample preparation.

Cryo-EM sample preparation and image acquisition

3 μL of purified GPR139-JNJ-63533054-miniG$_{s/q}$-Nb35 or GPR139-JNJ-63533054-G$_i$-scFv16 complex sample was applied to a glow-discharged holey carbon grid (CryoMatrix Amorphous alloy film R1.2/1.3, 300 mesh), and vitrified by Vitrobot Mark IV (Thermo Fisher Scientific). The chamber of Vitrobot was set to 100% humidity at 4 °C. Cryo-EM images were collected on a Titan Krios microscope operated at 300 kV equipped with Gatan K2 summit direct electron camera (Gatan) with a Gatan Quantum energy filter. Movies were taken in EFTEM nanoprobe mode, with 50 mm C2 aperture, at a calibrated magnification of 130,000 corresponding to a magnified pixel size of 1.04 Å. Each movie was comprised of 40 frames with a total dose of 60 electrons per Å^2, exposure time was 8.0 s with the dose rate of 8 e$^-$/Å^2/s. Data were collected by SerialEM software[4] with a defocus range of -1.0 to -2.0 μm.

3 μL of purified GPR139-JNJ-63533054-miniG$_{s/q}$-Nb35-GDP or GPR139-JNJ-63533054-miniG$_{s/q}$(I372A)-Nb35-GTP complex sample was applied to a glow-discharged holey carbon grid (CryoMatrix Amorphous alloy film R1.2/1.3, 300 mesh), and vitrified by Vitrobot Mark IV (Thermo Fisher Scientific). The chamber of Vitrobot was set to 100% humidity at 4 °C. Cryo-EM images were collected on a Titan Krios microscope operated at 300 kV equipped with Gatan K3 summit direct electron camera (Gatan) with a Gatan Quantum energy filter. Movies were taken in EFTEM nanoprobe mode, with 70 mm C2 aperture, at a calibrated magnification of 105,000 corresponding to a magnified pixel size of 0.832 Å. Each movie was comprised of 40 frames with a total dose of 60 electrons per Å^2, exposure time was 2.0 s with the dose rate of 20 e$^-$/Å^2/s. Data were collected by SerialEM software[4] with a defocus range of -1.2 to -2.0 μm.

Cryo-EM data processing and 3D reconstruction

For the GPR139-JNJ-63533054-miniG$_{s/q}$-Nb35 complex, 7,483 movies were collected and analyzed with cryoSPARC v.2.18 [5]. Beam-induced motion correction was performed using patch motion correction. Contrast transfer function (CTF) parameters for each dose-weighted micrograph were estimated by patch CTF estimation in cryoSPARC. A total of 6,082,524 particles were autopicked and used in two cycles of 2D classification. 1,149,703 particle projections were then selected to construct initial models and used as initial reference models for the subsequent 3D classification in cryoSPARC. The final dataset of 110,337 particle projections from the best class was further applied for final homogenous refinement, and a density map was obtained with a nominal resolution of 3.2 Å (determined by gold standard Fourier shell correlation (FSC) using the_0.143 criterion). Estimation of local resolution was performed with Local Resolution Estimation in cryoSPARC.

For the GPR139-JNJ-63533054-G$_i$-scFv16 complex, a total of 10,747 movies were collected and 807,155 particles were extracted for further 2D classification, and four different conformational subclasses were obtained by 3D homogenous refinement. Finally, 250,483 particle projections of the best class were further used for final homogenous refinement in cryoSPARC and the best density map was obtained with a nominal

resolution of 3.2 Å. Automatic local sharpening in DeepEMhancer [6] were performed for both EM density maps to optimize local density.

For the GPR139-JNJ-63533054-miniG$_{s/q}$-Nb35-GDP complex, a total of 7,908 movies were collected and 251,880 particles were extracted for 2D classification, and four different conformational subclasses were obtained by 3D homogenous refinement. Finally, 129,015 particle projections of the best class were further used for final homogenous refinement in cryoSPARC and the best density map was obtained with a nominal resolution of 3.7 Å.

For the GPR139-JNJ-63533054-miniG$_{s/q}$(I372A)-Nb35-GTP complex, 6,252 movies were collected and 603,396 particles were extracted for further 2D classification, and four different conformational subclasses were obtained by 3D homogenous refinement. Finally, 67,676 particle projections of the best class were further used for final homogenous refinement in cryoSPARC and the best density map was obtained with a nominal resolution of 3.3 Å. Automatic local sharpening in DeepEMhancer[6] were performed for all EM density maps to optimize local density.

Model building and structure refinement

For the GPR139-JNJ-63533054-miniG$_{s/q}$-Nb35 or GPR139-JNJ-63533054-G$_i$-scFv16 complexes, the miniGs399 protein from the GPR52-miniGs complex structure (PDB: 6UP7) [7] and the G$_i$ protein from the CB2-G$_i$ complex structure (PDB: 6KPF) [8], as well as the crystal structure of nociceptin/orphanin FQ peptide receptor (NOP) (PDB: 5DHG) [9] were used as starting models for model building and refinement against the electron density map. For the GPR139-JNJ-63533054-miniG$_{s/q}$-Nb35-GDP and GPR139-JNJ-63533054-miniG$_{s/q}$(I372A)-Nb35-GTP complexes, the solved nucleotide free miniG$_{s/q}$ coupled GPR139 structure was used for model building. The cryo-EM model was docked into the electron microscopy density map using Chimera [10], followed by iterative manual adjustment and rebuilding in Coot[11] and refinement using phenix.real_space_refine in PHENIX. The model statistics were validated using MolProbity[12]. Chimera and PyMOL (http://www.pymol.org) were using for structural figure preparation. The final refinement statistics are provided in Supplementary Table 5.

Bioluminescence resonance energy transfer (BRET) assay

Receptor mutations were generated as described in[13]. Briefly, mutations were introduced into N-terminally c-myc-tagged WT human GPR139 in the pEGFP-N1 vector (BD Biosciences). The myc tag of WT GPR139 does not change the receptor's pharmacology, as shown previously[13]. All mutagenesis and plasmids preparation were carried out by Genscript (USA). HEK293T cells were maintained in Dulbecco's Modified Eagle Medium (DMEM) supplemented with 10 % dialyzed fetal bovine serum (dFBS), penicillin (100 U/mL), and streptomycin (100 μg/mL) (Gibco). Cells were transfected by reverse transfection with GPR139 (1.2 ng, 2.4 ng, or 18.9 ng with WT or mutants), Venus 1-155 Gγ_2 (6.3 ng), Venus 159-239 Gβ_1 (6.3 ng), masGRK3ct-

NanoLuc (6.3 ng), and $G\alpha_q$ (12.6 ng) or $G\alpha_{i1}$ (6.3 ng) using Lipofectamine LTX and PLUS Reagent (375 nL of each). G proteins and BRET biosensors were a kind gift from the Martemyanov lab[14]. The total amount of transfected DNA was normalized to 150 ng with pcDNA3.1. All quantities are per well. A transfection solution was made by mixing in a 1:1 solution of DNA in OptiMEM (Gibco) with a solution of Lipofectamine LTX and PLUS Reagent (Invitrogen) in OptiMEM. 15 µL of transfection solution and 100 µL cell suspension containing 60,000 cells were added to each well of the microplate and incubated for 18-20 h before assays. The plates were coated with poly-D-lysine (Sigma, 4 mg/ml stock in PBS, diluted to final 40 µg/ml in PBS) prior to use. The stoichiometry of Gα and biosensors has been optimized previously[14]. WT GPR139 was transfected in three different amounts in the functional assay representing low, medium and normal/high expression of the receptor, as determined by the ELISA experiments.

Functional testing was performed by measuring agonist-induced BRET between Venus $G\beta_1\gamma_2$ and masGRK3ct-nLuc to examine the G protein signaling of GPR139 mutants under the presence of $G\alpha_q$ or $G\alpha_{i1}$, as described previously[14]. Briefly, prior to the experiment, the NanoGlo luciferase assay substrate, furimazine (Promega), was prepared by mixing 1 volume of the manufacturers stock solution with 250 volumes of BRET buffer (PBS (Gibco) + 0.5 mM $MgCl_2$ + 0.1% glucose). The agonist (JNJ-63533054 ([2-[(3-chlorophenyl) formamido]-N-[(1S)-1-phenylethyl] acetamide; CAS: 1802326-66-4]), Lundbeck compound 1a ([2-(3,5-Dimethoxybenzoyl)-N-(1-naphthyl)-hydrazinecarboxamide; CAS: 444932-31-4]), and Takeda compound 41 ([2-(4-oxo-3,4-dihydro-1,2,3-benzotriazin-3-yl)-N-[(1S)-1-[4-(trifluoromethoxy) phenyl] ethyl] acetamide; CAS: 1929519-13-0]) solutions were prepared in BRET buffer from DMSO stocks to a final DMSO concentration of 0.1 %. The cells were washed once with BRET buffer. Hereafter, luciferase substrate and agonists were added. Measurements were performed using LUMIstar Omega plate reader at room temperature. BRET ratios were determined by the ratio of emitted light by Venus (535 nm with a 30 nm band path width) to nLuc (475 nm with a 30 nm band path width). The basal BRET ratio (vehicle) was subtracted from the absolute BRET ratio at a given concentration and the responses were normalized to the maximal WT response with a compound. Mutants of low (0-50% of WT (18.9 ng)), medium (51-80% of WT (18.9 ng)), or normal/high (above 80% of WT (18.9 ng)) expression were normalized to low (1.2 ng), medium (2.4 ng), or normal/high (18.9 ng) expressed WT, respectively. Using this method, the impact of changed surface expression in a mutation on the observed absolute responses can be circumvented, because the response of the mutant is be expressed relative to the response of the wildtype of comparable surface expression. Data were fitted to the sigmoidal log (agonist) versus response function with variable slope using GraphPad Prism.

ELISA

The expression of GPR139 WT and mutants was tested with ELISA using receptors that were c-myc tagged. Indirect ELISA was performed using mouse primary antibody against the c-myc tag and goat secondary

detection antibody conjugated to horseradish peroxidase against the primary antibody. Cells were washed with Ca-PBS (PBS + 1 mM $CaCl_2$) and fixated (4% Paraformaldehyde (Sigma) in PBS) for 5 min). Hereafter, the cells were washed twice with Ca-PBS and incubated for 5 min with Triton X-100 (Sigma) for testing total expression, or Ca-PBS for testing surface expression. After washing twice with Ca-PBS, the wells were blocked for 30 min with blocking solution (3% Bovine Serum Albumin Fraction V (BSA) (Sigma) and 50 mM Trizma (Sigma) in Ca-PBS). Receptor was then labelled with primary antibody (mouse anti-c-myc (Invitrogen) and a 1:2000 dilution of blocking solution) for 45 min followed by one wash with blocking solution and two with Ca-PBS. Then secondary antibody (goat anti-mouse-HRP conjugated (Dako) and a 1:2000 dilution of blocking solution) was added. After 45 min, the cells were washed four times with blocking solution and four times with Ca-PBS. Subsequently, Ca-PBS was added to the wells followed by detection solution (SuperSignal ELISA Femto (ThermoFisher), a 1:1 mixture of Femto Stable Peroxidase Solution, and Femto Luminol Enhancer Solution). The resulting chemiluminescence was measured using an EnSpire plate reader. All incubations were performed at room temperature. The surface expression and total expression data were normalized to surface expressed and totally expressed WT GPR139 (18.9 ng), respectively.

Intracellular calcium mobilization assay

CHO K1 cells were seeded at a density of 100,000 cells in 10 cm dishes overnight at 37 °C in F-12 (Gibco) supplemented with 10% FBS. On the day of transfection, 1 µg plasmid DNA of GPR139 WT or its mutants were transfected to the cells by TransIT2020 (Mirus Bio). After 24 h, cells were trypsinized and seeded in black-sided, clear-bottom 384-well plates (Greiner Bio-one) at a density of 15,000 cells per well. On the day of assay, growth medium was removed, and cells were loaded with 20 µL/well of 1x Fluo-4 Direct Calcium dye (prepared in HBSS buffer) (Invitrogen) and incubated for 1 h at 37 °C in the dark. The FLIPR was programmed to take 10 readings (1 read per second), initially as a baseline before the addition of 10 µl of 3x JNJ-63533054 solution (prepared in HBSS buffer with 0.1% BSA). The fluorescence intensity was recorded for 2 min after addition of drug for agonist activity detection. Data were analyzed by nonlinear regression using GraphPad Prism 8.0.

Computational studies

Docking was performed with Glide[15-19] using XP precision and expanded sampling for the selection of initial poses. Different binding poses of JNJ-63533054 as well as two conformations of W170 in GPR139 in complex with G_i (2 initial ligand poses × 2 initial W170 conformations) and G_q (2 initial ligand poses) were also performed, each with three independent runs of 2 µs (i.e., 12 simulations with G_i and 6 with G_q). All protein-membrane systems were built with the CHARMM-GUI Membrane Builder[20,21]. The protein was modelled with the ff14SB protein force field[22], ligands with the GAFF2 force field[23], and lipids with the AMBER

Lipid17 force field. The details of these MD simulations are shown in previous work[8]. Figures were made with Chimera, PyMOL and Chimera X[24].

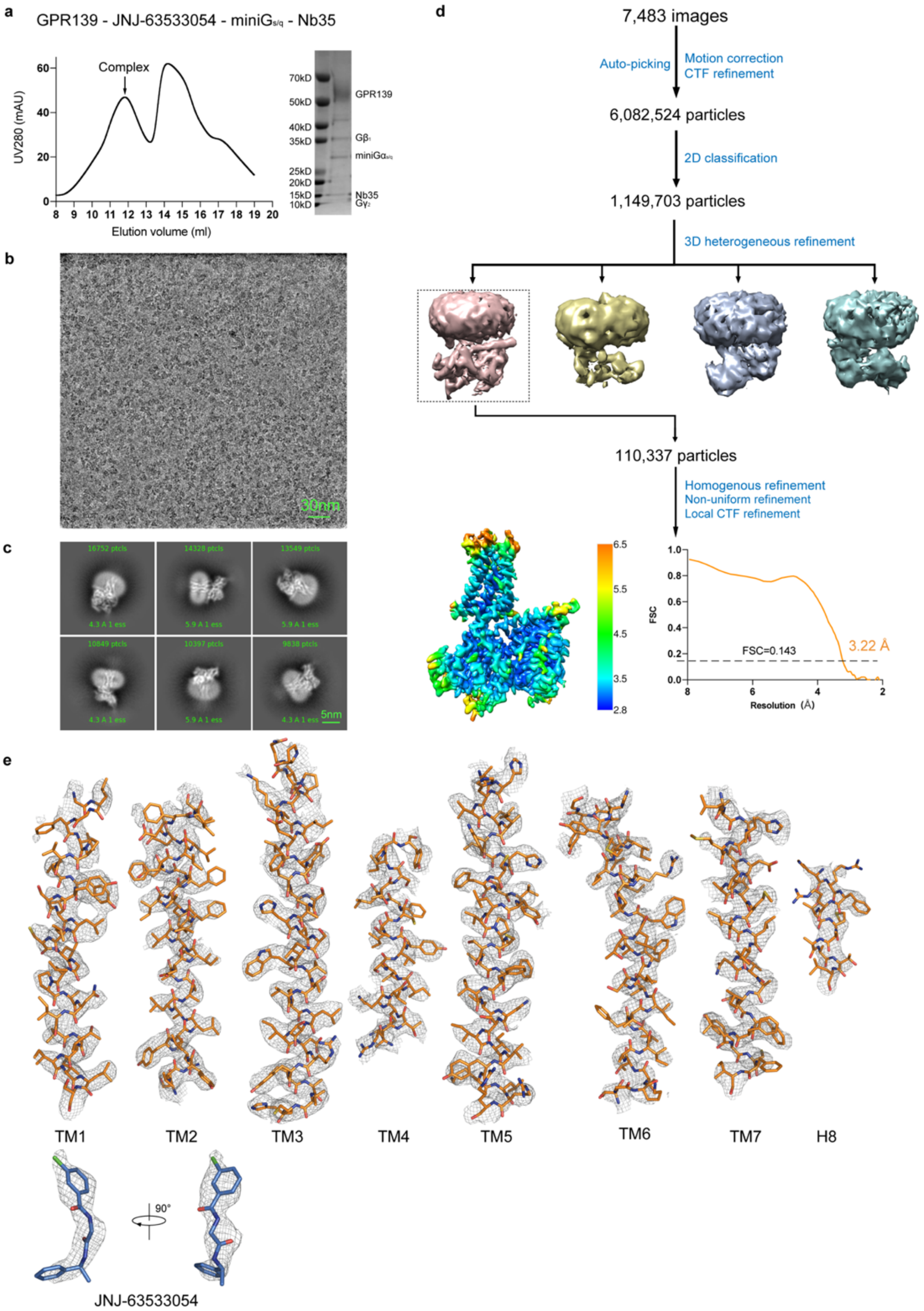

Fig. S1 Sample preparation and cryo-EM data processing of the GPR139-JNJ-63533054-miniG$_{s/q}$-NB35 complex

a. Superdex200 size-exclusion chromatography profile of GPR139-JNJ-63533054-miniG$_{s/q}$-NB35 complex and SDS-PAGE analysis of the purified complex.

b - **c**. Representative cryo-EM image (scale bar, 30 nm) from 7,485 movies (**b**) and 2D classification (scale bar, 5 nm) (**c**) of the complex.

d. Cryo-EM data processing flow chart, final local resolution for the density, and the 'gold-standard' FSC curve of GPR139-JNJ-63533054-miniG$_{s/q}$-NB35.

e. Cryo-EM density maps of transmembrane helixes, helix 8, and ligand JNJ-63533054.

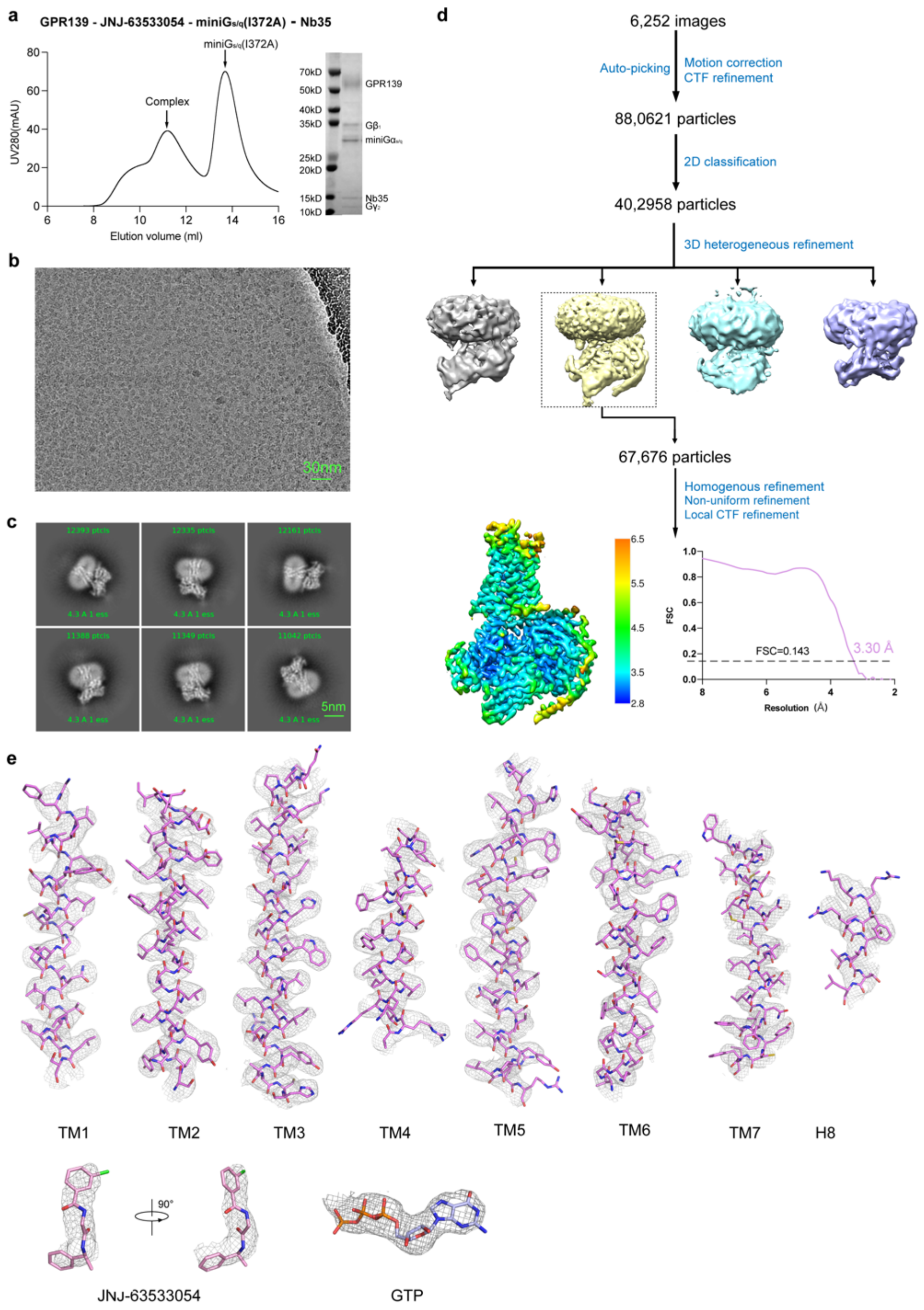

Fig. S2 Sample preparation and cryo-EM data processing of the GPR139-JNJ-63533054-miniG$_{s/q}$(I372A)-GTP-NB35 complex

a. Superdex200 size-exclusion chromatography of the GPR139-JNJ-63533054-miniG$_{s/q}$(I372A)-NB35 complex and SDS–PAGE analysis of the purified complex.

b - **c**. Representative cryo-EM image (scale bar, 30 nm) from 6,252 movies (**b**) and 2D classification (scale bar, 5 nm) (**c**) of the complex.

d. Cryo-EM data processing flow chart, final local resolution for the density, and the 'gold-standard' FSC curve of GPR139-JNJ-63533054--miniG$_{s/q}$(I372A)-GTP-NB35.

e. Cryo-EM density maps of each transmembrane helixes, pose3 of ligand JNJ-63533054 and GTP.

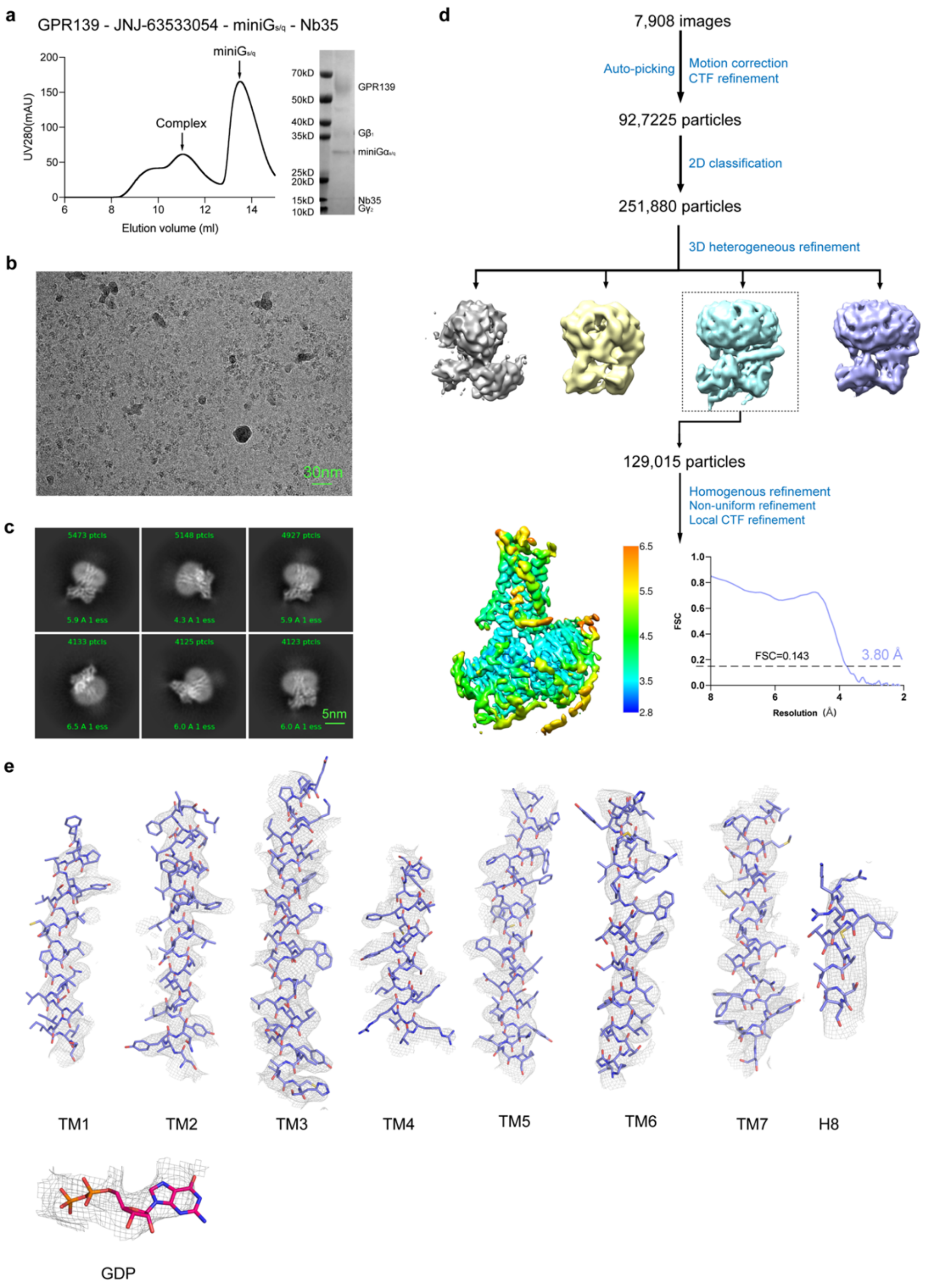

Fig. S3 Sample preparation and cryo-EM data processing of the GPR139-JNJ-63533054- miniG$_{s/q}$-GDP-NB35 complex

a. Superdex200 size-exclusion chromatography of the GPR139-JNJ-63533054-miniG$_{s/q}$-NB35 complex and SDS–PAGE analysis of the purified complex.

b - **c**. Representative cryo-EM image (scale bar, 30 nm) from 7,908 movies (**b**) and 2D classification (scale bar, 5 nm) (**c**) of the complex.

d. Cryo-EM data processing flow chart, final local resolution for the density, and the ‘gold-standard’ FSC curve of GPR139-JNJ-63533054--miniG$_{s/q}$-GDP-NB35.

e. Cryo-EM density maps of each transmembrane helixes and GDP.

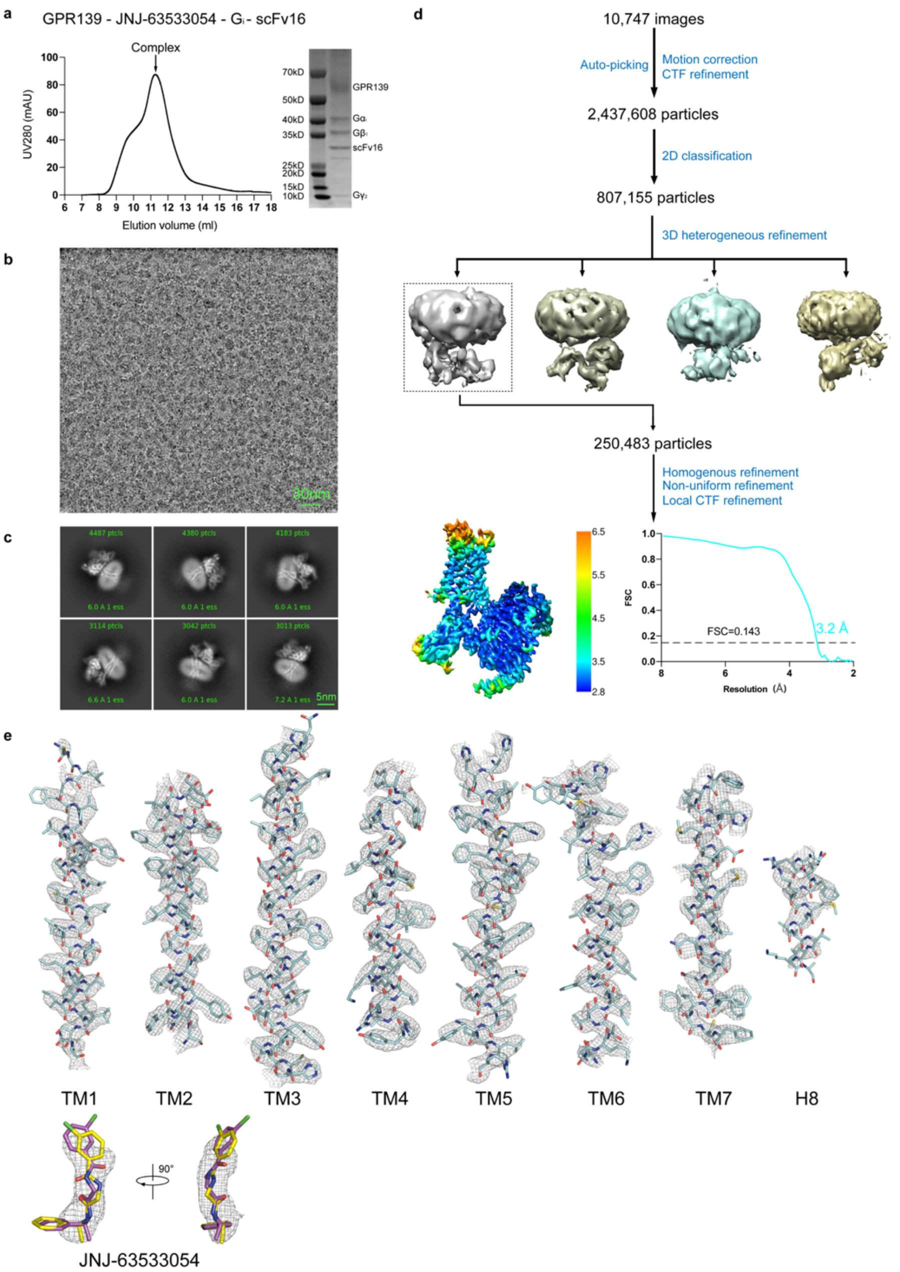

Fig. S4 Sample preparation and cryo-EM data processing of the GPR139-JNJ-63533054-G_i-scFv16 complex

a. Superdex200 size-exclusion chromatography of the GPR139-JNJ-63533054-G_i-scFv16 complex and SDS–PAGE analysis of the purified complex.

b - c. Representative cryo-EM image (scale bar, 30 nm) from 10,747 movies (**b**) and 2D classification (scale bar, 5 nm) (**c**) of the complex.

d. Cryo-EM data processing flow chart, final local resolution for the density, and the 'gold-standard' FSC curve of GPR139-JNJ-63533054-G_i-scFv16.

e. Cryo-EM density maps of each transmembrane helixes and two poses of ligand JNJ-63533054 (pose-1: yellow; pose-2: magenta).

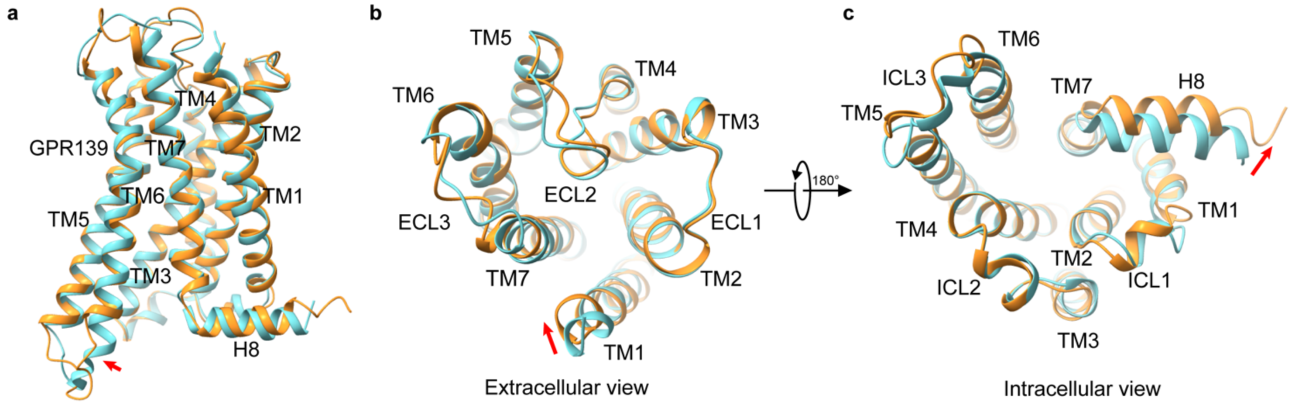

Fig. S5 Structure comparisons between miniG$_{s/q}$-coupled GPR139 and G$_i$-coupled GPR139.

a. Side view of the comparison between miniG$_{s/q}$-coupled GPR139 (orange) and G$_i$-coupled GPR139 structure (cyan) with a 1.25 Å root mean square deviation (RMSD) of the Cα atoms. The red arrows show the main conformational differences between the two structures.

b - **c**. Extracellular view (**b**) and intracellular view (**c**) of the comparison between miniG$_{s/q}$-coupled GPR139 and G$_i$-coupled GPR139.

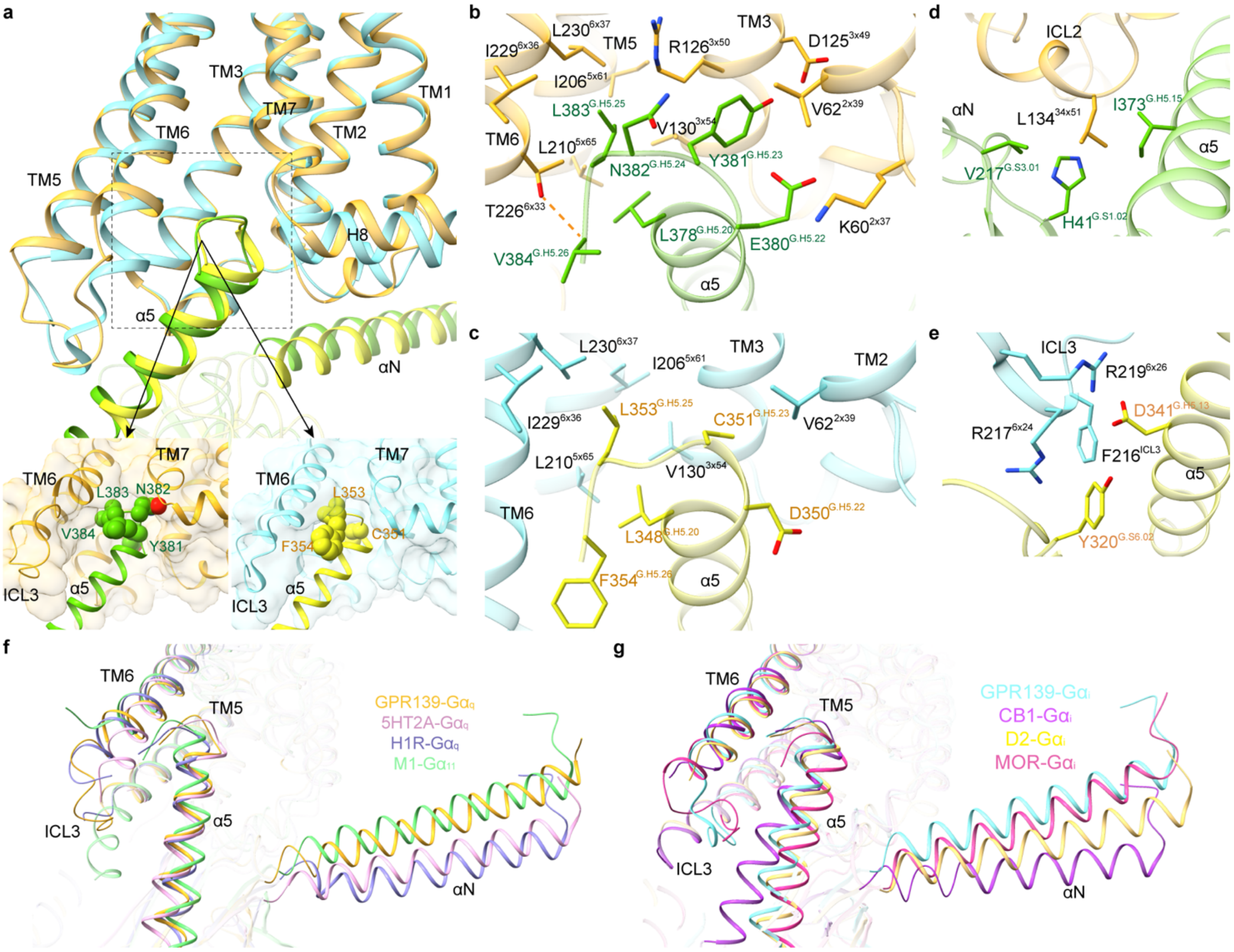

Fig. S6 Comparison of GPR139-miniG$_{s/q}$ and GPR139-G$_i$ binding interfaces and the G-protein interface comparison between GPR139 and other GPCR-G$_q$/G$_i$ complexes.

Despite the similarity in their overall binding interface, the miniG$_{s/q}$- and G$_i$-bound GPR139 structures exhibit different detailed molecular interaction patterns across both the α5 helices of the Gα proteins and the intracellular loops of the receptor, which can contribute to the selective engagement of G proteins. Though the α5 helices of Gα_i and Gα_q adopt similar orientations, Y381$^{G.H5.23}$ and N382$^{G.H5.24}$ from Gα_q interact with TM3 and TM7 in GPR139, while Gα_i lacks such interactions because the residues in the corresponding positions, C351$^{G.H5.23}$ and G352$^{G.H5.24}$ have shorter sidechains. Additional differences are found in the receptor ICL2-3. In the GPR139-G$_i$ structure, ICL3 is more structurally ordered, has a better EM map, and packs closer to the α5 helix than in the Gα_q complex. The residues F216^{ICL3}, R217^{6x24} and R219^{6x26} are, therefore, better situated for interacting with Gα_i than Gα_q. Though the conformation of ICL2 in the two structure complexes are almost identical, the αN-β1 junction and the β2-β3 loop of Gα_q in the GPR139-miniG$_{s/q}$ complex are closer to the receptor ICL2.

a. Comparison of the α5 helices in Gα and receptors between GPR139-miniG$_{s/q}$ (orange - green) and GPR139-G$_i$ (cyan - yellow) structures when aligned on the receptors. The binding pocket for the Gα$_{s/q}$-α5 and Gα$_i$-α5 C termini are also shown.

b - **c**. Close up view (of dashed box in A) showing the hydrophobic binding core of GPR139-miniGα$_{s/q}$ (**b**) and GPR139-Gα$_i$ (**c**). V384$^{G.H5.26}$ in Gα$_q$ is hydrogen-bonded by T226^{6x33} in GPR139. Y381$^{H5.23}$ and E380$^{H5.22}$ in Gα$_q$ form polar interactions with D125^{3x49}, R126^{3x50}, V62^{2x39} and K60^{2x37} in GPR139.

d. In the GPR139-miniG$_{s/q}$ structure, L134^{34x51} in ICL2 forms interaction with αH5, S1 and S3 in Gα$_{s/q}$.

e. Interactions between ICL3 and Gα$_i$ in the GPR139-G$_i$ structure.

f. Superposition of GPR139-miniGα$_{s/q}$ complex (orange) with H1R-Gα$_q$ (purple, PDB: 7DFL) M$_1$R-Gα$_{11}$ (green, PDB: 6OIJ), and 5-HT$_{2A}$-miniGα$_q$ (pink, PDB: 6WHA) complexes. Comparison of our G$_q$-bound structures of GPR139, serotonin 5-HT$_{2A}$[25], and histamine H$_1$[26] receptors, shows that the C-terminal α5 helices have similar conformations. In contrast, their N-terminal αN helices differ substantially in their orientations relative to the receptors, due to their diverse interactions with ICL2-3 of the receptors. Furthermore, comparing these three structures to that of the muscarinic M$_1$-G$_{11}$ structure complex[27], its cytoplastic TM5 is more extended and forms additional hydrophobic interactions with Gα$_{11}$. As a consequence of the unique interactions, the insertion angles of the α5 helix differ by up to 8° in the four G$_q$-coupled receptors.

g. Superposition of GPR139-Gα$_i$ complex (cyan) with μ-Gα$_i$ (hotpink, PDB: 6DDE), D$_2$-Gα$_i$ (yellow, PDB: 7JVR), and CB1-Gα$_i$ (purple, PDB: 6KPG) complexes. The GPR139 and G$_{i1}$ binding mode is quite similar to that of previously solved class A GPCR-G$_{i/o}$ complex structures. The primary interaction interface is composed of TM3, TM5, TM6, ICL2 and ICL3 of the receptor and the α5 helix of the Gα$_i$.

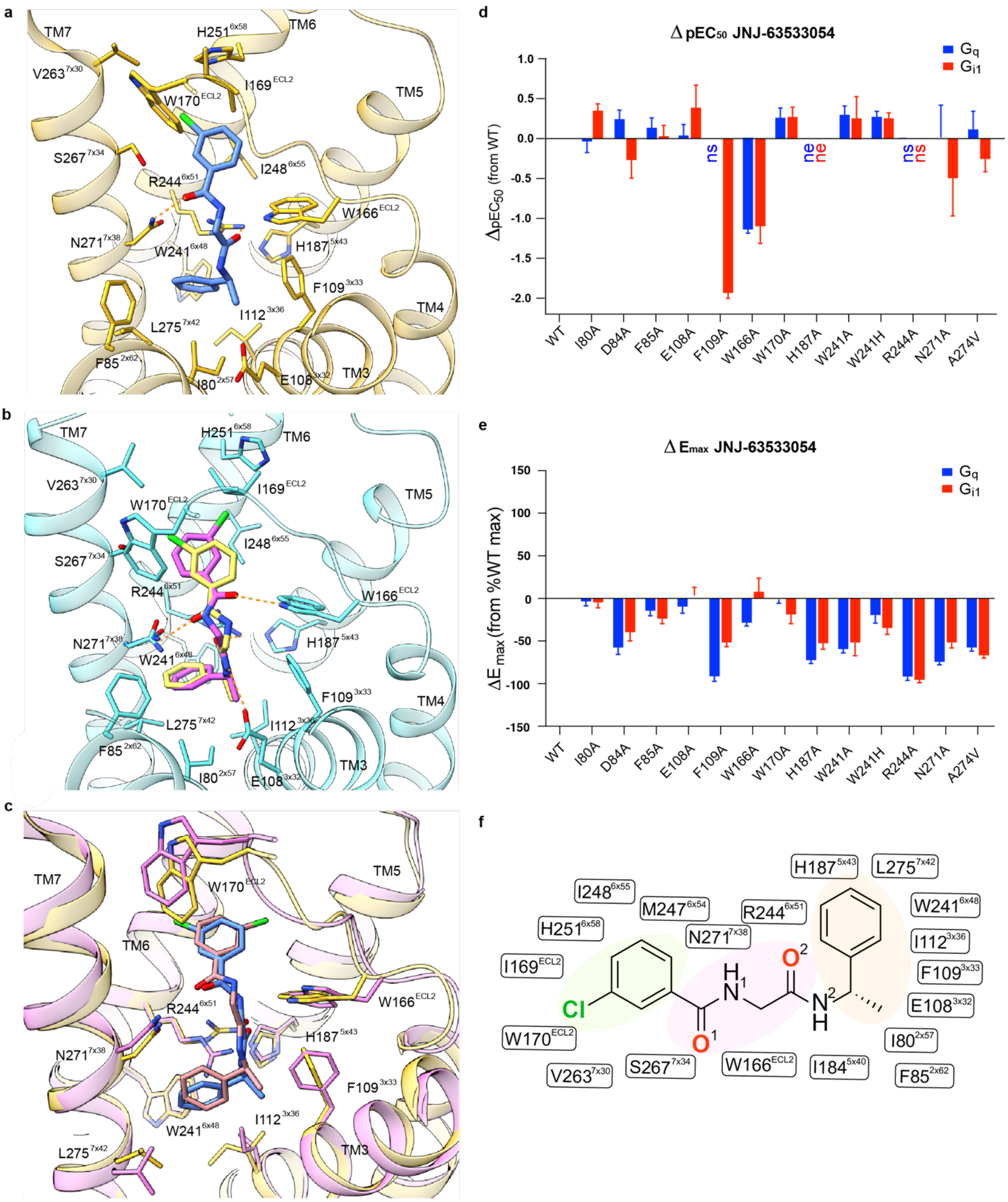

Fig. S7 Characterization of JNJ-63533054 binding in GPR139

a - **b**. Interactions of JNJ-63533054 with key residues of GPR139 in the GPR139-miniG$_{s/q}$ complex (**a**) and GPR139-G$_i$ complex (**b**). Residues are shown as sticks (orange in GPR139-miniG$_{s/q}$ complex structure and cyan in GPR139-G$_i$ complex structure) and the polar interactions between W166^{ECL2}, R244^{6x51}, N271^{7x38} and

JNJ-63533054 are shown with orange dotted lines. JNJ-63533054 in the GPR139-miniG$_{s/q}$ complex structure: blue. JNJ-63533054 in GPR139-G$_i$ complex structure: yellow (pose-1) and magenta (pose-2).

c. Ligand binding pocket comparisons between GTP bound and nucleotide free GPR139-miniG$_{s/q}$ complexes. JNJ-63533054 in GPR139-miniG$_{s/q}$-GTP complex structure: pink (pose-3). GPR139-miniG$_{s/q}$-GTP complex structure: light purple.

d - **e**. Effect of key residue mutations on the ligand potency (pEC$_{50}$) (**d**) and E$_{max}$ (**e**). ns: no signal; ne: no estimation. Data are mean ± s.e.m. (n = 3).

f. Schematic of JNJ-63533054's interactions with key residues in GPR139.

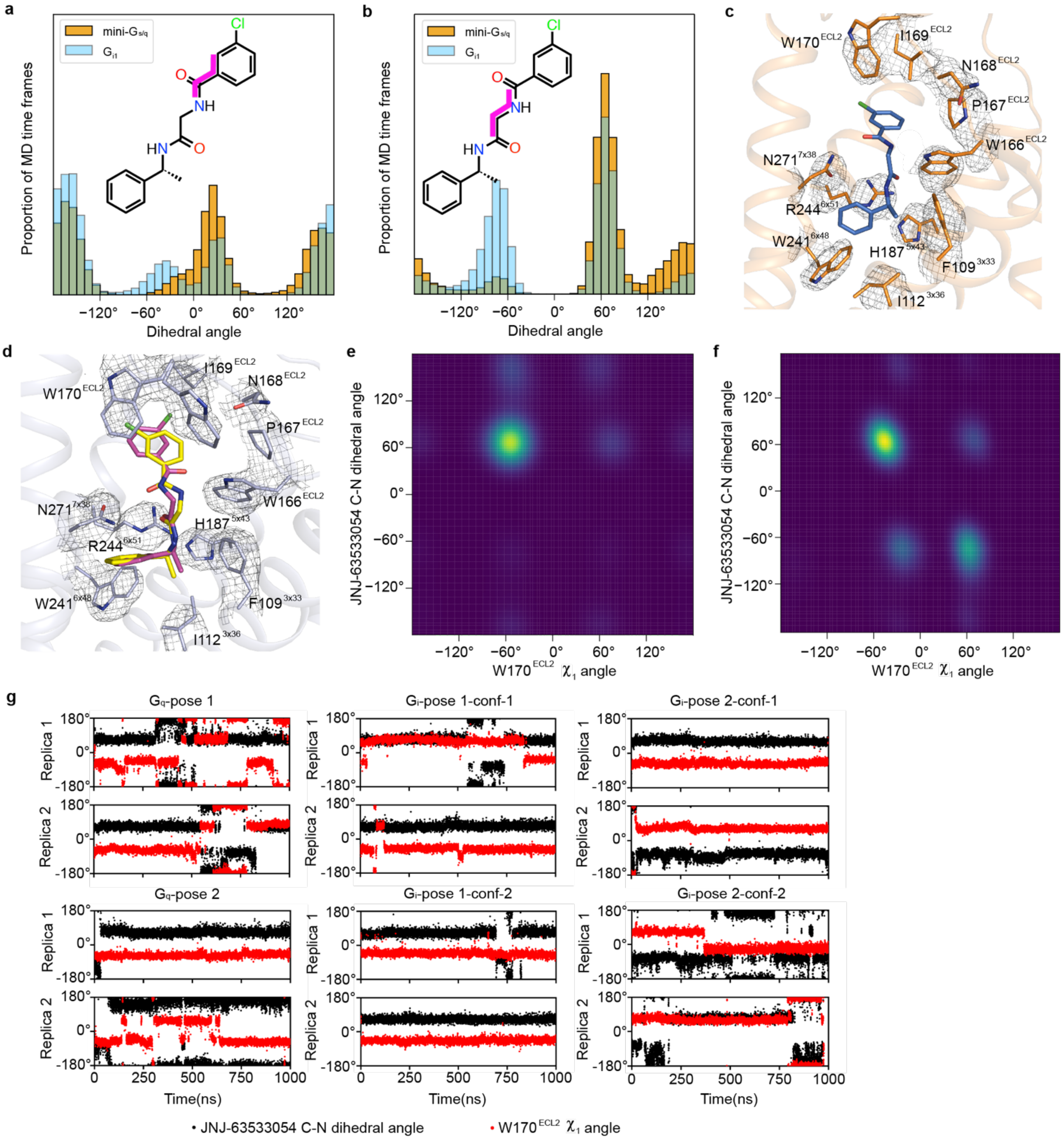

Fig. S8 Analysis of the dihedral angles distribution of ligand and W170.

a - b. Dihedral angle around the C-N bond between the two amide groups (**a**) and for the (O=) C-C (chlorophenyl) bond (**b**). Orange and blue bars show the distribution in the GPR139-miniG$_{s/q}$ and GPR139-G$_{i1}$ structures, and the green areas show overlapping peaks between the two simulations.

c - d. Density map of the key residues in ligand binding pocket and ECL2 residues in the GPR139-miniG$_{s/q}$ (**c**) and GPR139-G$_{i1}$ (**d**) structures

e - f. Density plots of the distribution of the JNJ-63533054 C-N dihedral angle with respect to the side chain dihedral χ_1 of W170^{ECL2} during the MD simulations with GPR139-miniG$_{s/q}$ (**e**) and GPR139-G$_i$ (**f**).

g. MD simulation curves of JNJ-63533054 C-N dihedral angle and W170^{ECL2} χ_1 angle. Black dots represent the results of JNJ-63533054 C-N dihedral angle. Red dots represent the results of W170^{ECL2} χ_1 angle.

a

6x51 AAs	Occurence	6x51 AAs	Occurence
Y	35.4%	N	2.4%
F	29.9%	E	1.4%
L	10.8%	C	1.0%
I	5.2%	G	1.0%
V	3.8%	M	1.0%
H	3.1%	R	1.0%
S	3.1%	Other	<1%

b

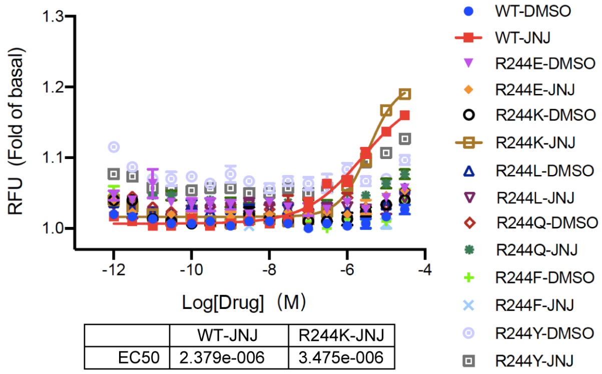

c

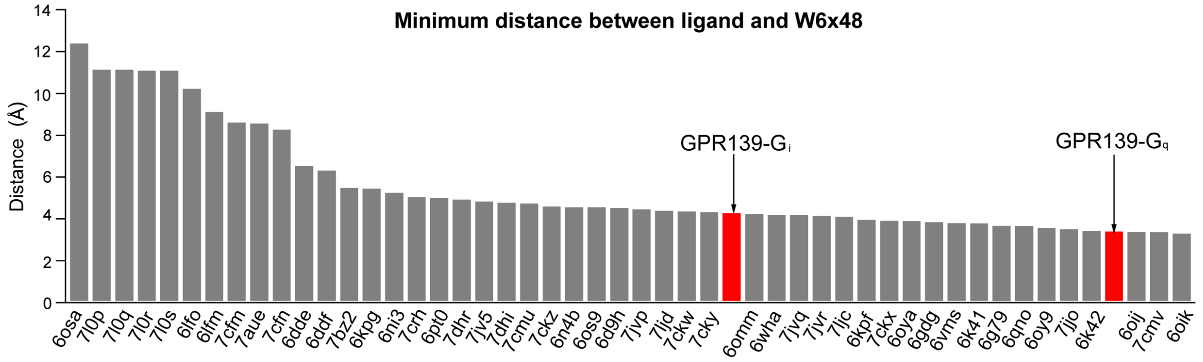

d

7x50 AAs	Occurence
P	94.1%
A	2.1%
L	0.7%
C	0.7%
T	0.7%
V	0.7%
M	0.3%
F	0.7%

e

	7x49	7x50	7x51	7x52	7x53	7x54	7x55
GPR139	N	F	F	L	Y	C	F
GPR142	N	F	G	L	Y	C	F
MC_4	D	P	L	I	Y	A	L
A_{2A}	N	P	F	I	Y	A	Y
β_2AR	N	P	L	I	Y	C	R
D_2	N	P	I	I	Y	T	T
μ	N	P	V	L	Y	A	F
CXCR2	N	P	L	I	Y	A	F
CB1	N	P	I	I	Y	A	L
5-HT2A	N	P	L	V	Y	T	L
GPR52	N	C	V	I	Y	S	L

f

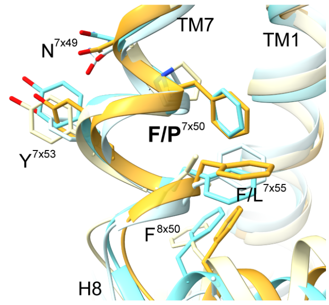

Fig. S9 Unique 6x51 and 7x50 sites analysis in GPR139.

a. Occurrence of different amino acids at the 6x51 site in 288 class A GPCRs. Three GPCRs have R^{6x51}: y_6 (only has 6 TMs), GPR139, and GPR142.

b. Ca^{2+} signal of different $R244^{6x51}$ mutation responses to JNJ-63533054. Data are mean ± s.e.m. (n = 3).

c. Minimum distance between ligand and "toggle switch" W^{6x48} of all solved agonist and G protein bound class A receptor cryo-EM structures.

d. Occurrence of different amino acids in the 7x50 site in 288 class A GPCRs. Only GPR139 and GPR142 own an F in 7x50.

e. Sequence alignment of the NPxxY motif in GPR139, GPR142, MC_4 $A_{2A}R$, β_2AR, D_2, μ-opioid, CXCR2, CB1, 5-HT_{2A}, and GPR52. Conserved residues are colored in orange and unique F7.50 in GPR139 and GPR142 are colored in green.

f. The "NPxxY" motif comparison between the activated GPR139 (miniG$_{s/q}$ coupled: orange; G_i coupled: cyan), M_1 (G_{11} coupled, PDB: 6OIJ; light yellow) and μ-opioid (G_i coupled, PDB: 6DDE; light cyan) receptors.

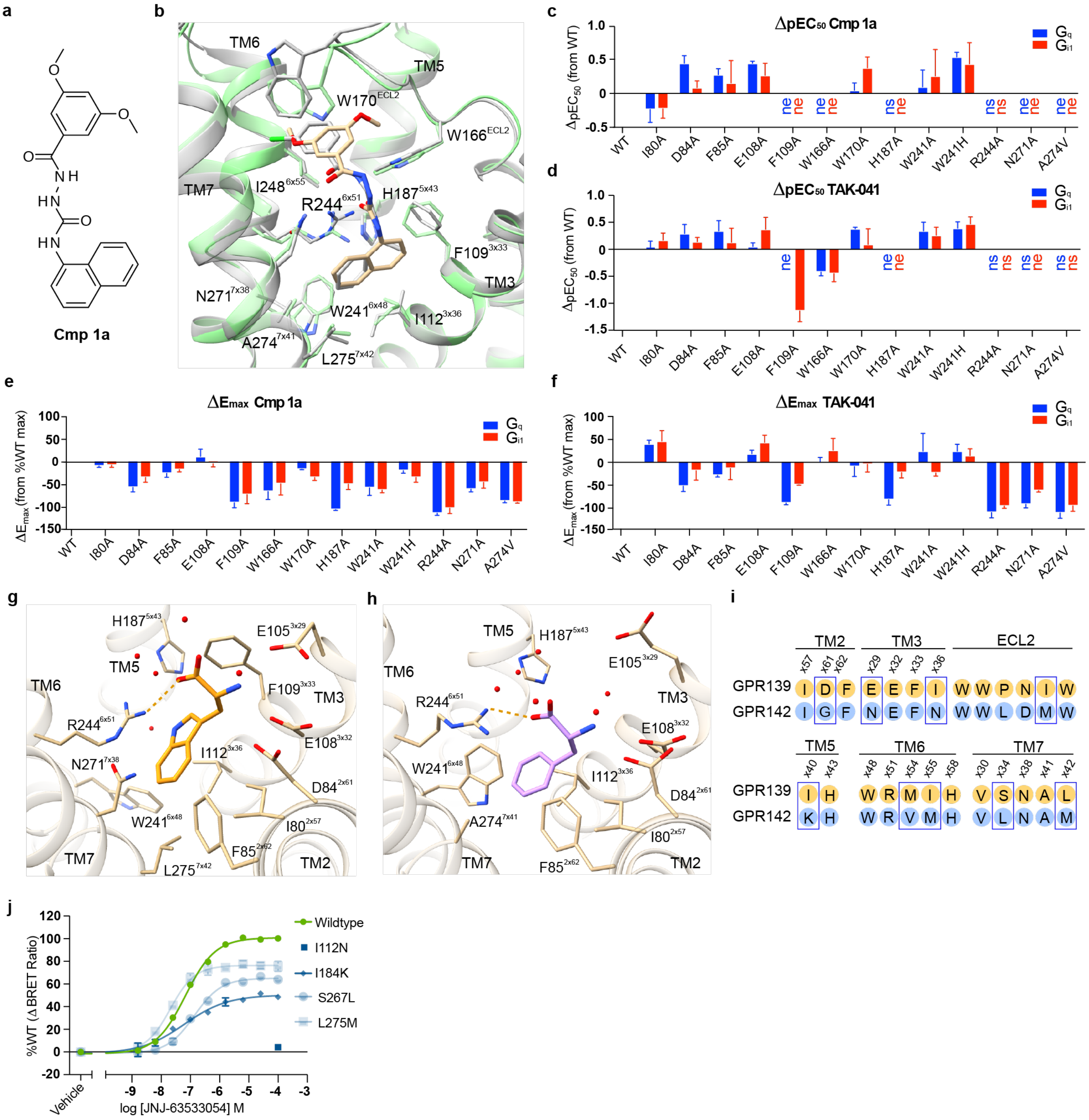

Fig. S10 Docking poses of representative surrogate ligands Cmp 1a, L-Trp and L-Phe to GPR139 and the selectivity hotspot in GPR139's homolog GPR142

a. Chemical structures of GPR139 agonists Cmp 1a (Lundbeck).

b. The comparison of Cmp 1a docking pose in GPR139 (green) and JNJ-63533054 in GPR139-miniG$_{s/q}$ complex (gray).

c - f. BRET validation of key residues involved in TAK-041/Cmp 1a and receptor interactions. ns: no signal; ne: no estimation.

g - h. L-Trp (**g**) and L-Phe (**h**) docking pose in GPR139.

i. Sequence alignment of residues involved in ligand binding between GPR139 and GPR142. Different

mutations in the ligand binding site are highlighted by the blue box.

j. Effects of mutants (mentioned in **i**) on GPR139 in JNJ63533054-induced G_q coupling.

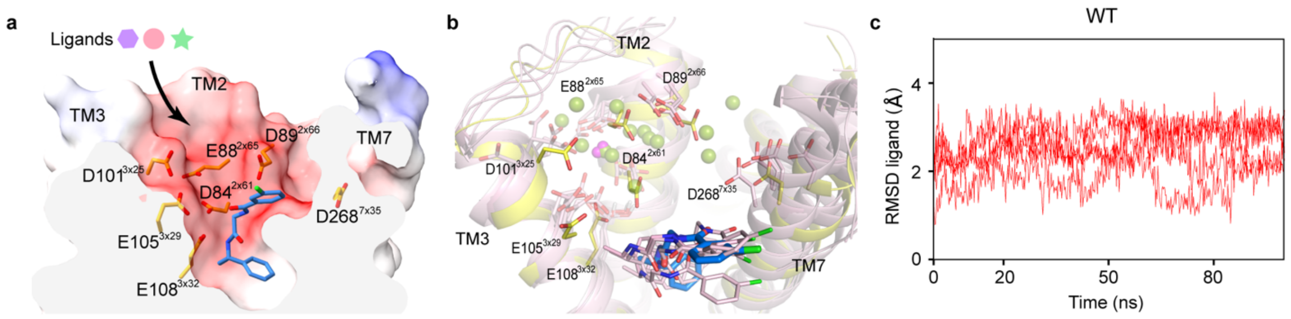

Fig. S11 Analysis of negative charged residues near the ligand binding pocket and WT GPR139 molecular dynamic simulation

a. Electrostatic surface presentation of GPR139 shows a negatively charged wall in, and near the JNJ-63533054 binding pocket.

b. Cryo-EM structure (yellow) and representative snapshots (light pink) from five 100-ns MD simulations with the WT GPR139-miniG$_{s/q}$ complex. Green and magenta spheres indicate positively charged Na^+ ions and Ca^{2+} ions, respectively.

c. Ligand RMSD value (with respect to the Cryo-EM structure) in WT simulations.

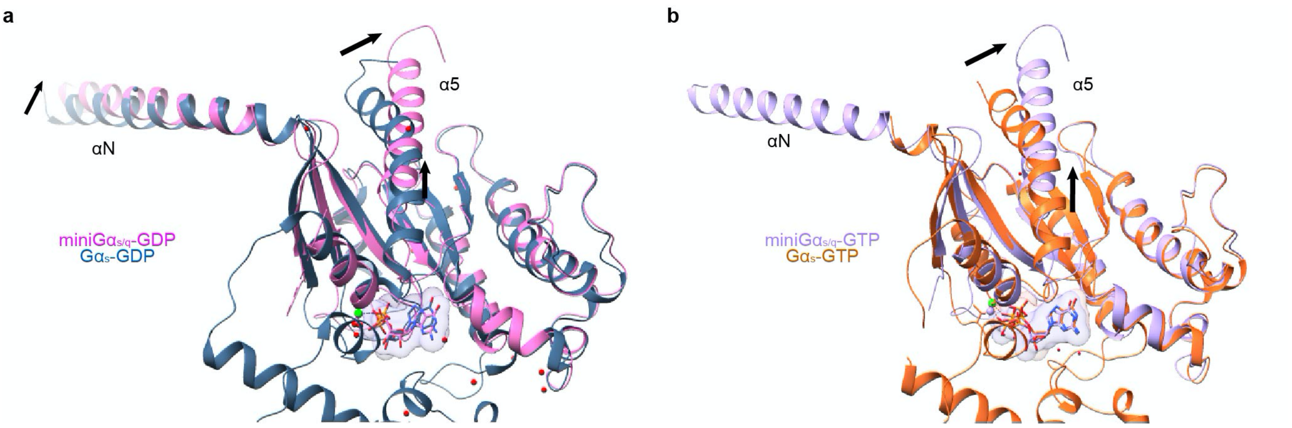

Fig. S12 Structural comparison of the nucleotide bound Gα$_{s/q}$ in GPR139-miniG$_{s/q}$ complexes and nucleotide bound Gα$_s$ crystal structures

a. The structure comparisons between GDP bound miniGα$_{s/q}$ in GPR139-miniG$_{s/q}$ complex and the crystal structure of GDP bound Gα$_s$ (PDB: 6EG8). Black arrows show the movements.

b. The structure comparisons between GTP bound miniGα$_{s/q}$ in GPR139-miniG$_{s/q}$ complex and the crystal structure of GTP bound Gα$_s$ (PDB: 1AZT). Black arrows show the movements.

Table S1. GPR139 WT and intracellular sodium ion site, structure construct, and TM7 proline kink mutations. Ligands from Jansen RD (JNJ-63533054), Takeda (TAK-041), and Lundbeck A/S (Cmp1a). The pharmacology of WT and mutant GPR139 expressed in HEK-293 cells was examined using a BRET G protein-coupling assay, which measures the dissociation of the G protein heterotrimer [14]. GPR139 mutant responses are normalized to the WT of a comparable expression category, as explained in detail in the **Methods** section. Surface expression is shown as percentage of 18.9 ng WT. E_{max} is shown as a percentage of respective WT control according to the expression category. For mutants where complete CRCs were not obtained (No estimation), E_{max} represents the response at the highest tested concentration – 100 µM for **JNJ**, 30 µM for **041**, or 30 µM for **1a**. Data represent mean ± SD of at least three independent experiments. Mutants that produced a signal below 10% of WT (No Signal) were tested twice. Asterisk (*) indicates significant differences compared to respective WT control; $p < 0.05$, determined by one-way ANOVA with Dunnett's post hoc test.

GPR139 res. pos.	GPCRdb generic residue number	WT AA	Mut AA	Rationale	Surface Expression	$G\alpha_q$ activation						$G\alpha_{i1}$ activation					
						JNJ-63533054		TAK-041		Cmp1a		JNJ-63533054		TAK-041		Cmp1a	
						pEC_{50}	E_{max}	pEC_{50}	E_{max}	pEC_{50}	E_{max}	pEC_{50}	E_{max}	pEC_{50}	E_{max}	pEC_{50}	E_{max}
-	-	-	-	WT reference construct (1.2 ng; Low Expressed)	39 ± 2	7.12 ± 0.12	86 ± 6	6.87 ± 0.41	105 ± 6	6.59 ± 0.22	99 ± 21	7.05 ± 0.18	87 ± 6	6.79 ± 0.21	93 ± 17	6.60 ± 0.18	105 ± 8
-	-	-	-	WT reference construct (2.4 ng; Medium Expressed)	75 ± 10	7.15 ± 0.17	97 ± 7	6.65 ± 0.09	100 ± 21	6.58 ± 0.14	114 ± 33	7.18 ± 0.15	108 ± 13	6.87 ± 0.11	104 ± 16	6.70 ± 0.18	107 ± 6
-	-	-	-	WT reference construct (18.9 ng; Normal/High Expressed)	124 ± 21	7.17 ± 0.11	101 ± 6	6.69 ± 0.31	105 ± 8	6.69 ± 0.05	114 ± 5	7.16 ± 0.11	95 ± 14	6.90 ± 0.19	90 ± 3	6.90 ± 0.17	110 ± 17
73	2x50	D	A	Na+ site in lower 7TM cavity	20 ± 6	No signal	-1 ± 1*					No signal	8 ± 1*				
115	3x39	S	A	Na+ site in lower 7TM cavity	91 ± 10	7.04 ± 0.27	57 ± 3*					7.05 ± 0.14	87 ± 10				
73 +115	2x50 +3x39	D +S	A +A	Na+ site in lower 7TM cavity, double mutant: D73A + S115A (to evaluate effect of total loss of IC Na^+ binding)	20 ± 8	No signal	4 ± 2*					No estimation	11 ± 5*				
62	2x39	S	V	Mutation used in the structure construct	110 ± 2	7.09 ± 0.31	55 ± 5*					7.23 ± 0.13	107 ± 5				
282	7x50	F	P	TM7 proline kink	91 ± 12	7.38 ± 0.33	35 ± 3*					No estimation	34 ± 13*				

Table S2. GPR139 ligand site mutations. JNJ-63533054, Cmp 1a, and TAK-041. See Table S1 legend for further information.

**GPR 139 res. pos.**	**GPCRdb generic residue number**	**WT AA**	**Mut AA**	**JNJ**	**41**	**1a**	**Surface Expression**	**Gα_q activation**						**Gα_{i1} activation**					
								**JNJ-63533054**		**TAK-041**		**Cmp1a**		**JNJ-63533054**		**TAK-041**		**Cmp1a**	
								**pEC$_{50}$**	**E$_{max}$**	**pEC$_{50}$**	**E$_{max}$**	**pEC$_{50}$**	**E$_{max}$**	**pEC$_{50}$**	**E$_{max}$**	**pEC$_{50}$**	**E$_{max}$**	**pEC$_{50}$**	**E$_{max}$**
80	2x57	I	A	JNJ		1a	153 ± 13	7.12 ± 0.14	89 ± 5	6.73 ± 0.12	143 ± 8*	6.36 ± 0.19*	99 ± 3	7.51 ± 0.09	90 ± 6	7.02 ± 0.13	142 ± 23*	6.50 ± 0.14	99 ± 5
84	2x61	D	A	JNJ		1a	30 ± 7	7.42 ± 0.12	35 ± 8*	6.99 ± 0.17	50 ± 12*	7.04 ± 0.11*	52 ± 10*	6.84 ± 0.23	55 ± 10*	6.99 ± 0.08	77 ± 21	6.81 ± 0.10	72 ± 11*
85	2x62	F	A	JNJ		1a	74 ± 12	7.32 ± 0.13	78 ± 6*	7.04 ± 0.19*	74 ± 4	6.87 ± 0.09	83 ± 9	7.19 ± 0.14	71 ± 6*	6.98 ± 0.26	82 ± 25	6.88 ± 0.33	89 ± 5*
108	3x32	E	A	JNJ		1a	89 ± 20	7.19 ± 0.14	83 ± 8*	6.75 ± 0.07	121 ± 8	7.04 ± 0.03*	120 ± 16	7.54 ± 0.28	101 ± 9	7.22 ± 0.22	139 ± 16*	6.99 ± 0.18	102 ± 7
109	3x33	F	A	JNJ	41	1a	98 ± 14	No signal	1 ± 6*	No estimation	13 ± 4*	No estimation	18 ± 11*	5.17 ± 0.07*	43 ± 5*	5.71 ± 0.20*	46 ± 1*	No estimation	34 ± 20*
166	ECL2	W	A	JNJ	41	1a	124 ± 35	5.98 ± 0.05	64 ± 4*	6.28 ± 0.07*	103 ± 10	No estimation	43 ± 18*	6.01 ± 0.22*	107 ± 16	6.40 ± 0.15*	122 ± 26	No estimation	58 ± 25*
170	ECL2	W	A	JNJ	41	1a	74 ± 10	7.44 ± 0.12	93 ± 4	7.08 ± 0.03*	93 ± 22	6.64 ± 0.11	92 ± 1	7.43 ± 0.12	76 ± 11*	6.94 ± 0.29	91 ± 17	7.10 ± 0.16	72 ± 7*
187	5x43	H	A	JNJ			33 ± 13	No estimation	20 ± 4*	No estimation	20 ± 12*	No signal	3 ± 2*	No estimation	42 ± 7*	No estimation	73 ± 12	No estimation	57 ± 12*
241	6x48	W	A	JNJ	41	1a	36 ± 3	7.48 ± 0.11	33 ± 4*	7.04 ± 0.16	127 ± 39	6.69 ± 0.25	51 ± 17*	7.42 ± 0.27	43 ± 15*	7.11 ± 0.15	72 ± 7	6.98 ± 0.39	44 ± 6*
241	6x48	W	H	JNJ	41	1a	153 ± 20	7.45 ± 0.07	73 ± 9*	7.09 ± 0.12*	127 ± 15*	7.13 ± 0.07*	89 ± 6*	7.41 ± 0.07	60 ± 7*	7.32 ± 0.13	110 ± 15	7.16 ± 0.31	72 ± 11*
244	6x51	R	A	JNJ	41	1a	22 ± 5	No signal	0,9 ± 4*	No signal	-8 ± 12*	No signal	-5 ± 5*	No signal	-0,6 ± 3*	No signal	-0,8 ± 5*	No signal	3.7 ± 12*
271	7x38	N	A	JNJ	41	1a	48 ± 15	7.16 ± 0.40	18 ± 3*	No signal	10 ± 8*	No estimation	48 ± 6*	6.61 ± 0.47	43 ± 6*	No estimation	33 ± 3*	No estimation	61 ± 13*
274	7x41	A	V	JNJ	41	1a	51 ± 3	7.29 ± 0.23	35 ± 4*	No signal	-9 ± 12*	No estimation	22 ± 4*	6.85 ± 0.16	28 ± 3*	No Signal	-0.3 ± 12*	No estimation	17 ± 2*

Table S3. Mutations of unique GPR139 ligand site residues not conserved in GPR142. See Table S1 legend for more information.

GPR 139 res. pos.	GPCRdb generic residue number	WT AA	Mut AA	JNJ	41	1a	Surface Expression	$G\alpha_q$ activation						$G\alpha_{i1}$ activation					
								JNJ-63533054		TAK-041		Cmp1a		JNJ-63533054		TAK-041		Cmp1a	
								pEC_{50}	E_{max}	pEC_{50}	E_{max}	pEC_{50}	E_{max}	pEC_{50}	E_{max}	pEC_{50}	E_{max}	pEC_{50}	E_{max}
105	3x29	E	A				118 ± 7	7.35 ± 0.15	106 ± 5	7.07 ± 0.07*	129 ± 2*	6.91 ± 0.09	127 ± 9	7.44 ± 0.14	113 ± 11	7.17 ± 0.05	143 ± 25*	7.40 ± 0.07*	139 ± 12
112	3x36	I	N	JNJ	41	1a	36 ± 4	No signal	4 ± 4*	No signal	7 ± 5*	No estimation	25 ± 8*	No estimation	42 ± 9*	No estimation	30 ± 6*	No estimation	39 ± 4*
169	ECL2	I	A	JNJ	41		98 ± 8	7.23 ± 0.08	83 ± 7*	6.66 ± 0.15	88 ± 7	6.73 ± 0.02	87 ± 4*	7.15 ± 0.18	102 ± 17	6.81 ± 0.21	103 ± 22	6.80 ± 0.26	100 ± 17
169	ECL2	I	M	JNJ	41		84 ± 29	7.60 ± 0.05*	92 ± 9	7.30 ± 0.04*	86 ± 8*	6.81 ± 0.08	69 ± 7*	7.82 ± 0.15*	103 ± 17	7.29 ± 0.16	79 ± 7	7.20 ± 0.22	82 ± 9
184	5x40	I	K				33 ± 7	7.22 ± 0.18	50 ± 5*	6.74 ± 0.13	100 ± 22	6.35 ± 0.12	60 ± 14*	6.98 ± 0.10	82 ± 5	6.78 ± 0.26	142 ± 33*	6.35 ± 0.14	75 ± 12*
247	6x54	M	A	JNJ			56 ± 6	7.47 ± 0.13	60 ± 5*	6.70 ± 0.13	70 ± 9	6.87 ± 0.23	68 ± 7*	7.39 ± 0.05	58 ± 12*	6.95 ± 0.34	62 ± 13*	6.98 ± 0.26	62 ± 5*
247	6x54	M	V	JNJ			88 ± 5	7.44 ± 0.01	76 ± 6*	6.89 ± 0.11	57 ± 4*	6.80 ± 0.14	84 ± 6*	7.70 ± 0.01*	84 ± 7	7.04 ± 0.21	60 ± 12	7.20 ± 0.22	79 ± 8
248	6x55	I	M	JNJ			121 ± 10	7.52 ± 0.11	86 ± 3*	7.12 ± 0.12*	105 ± 4	7.06 ± 0.14*	84 ± 9*	7.46 ± 0.16	89 ± 1	7.27 ± 0.06	123 ± 12	7.41 ± 0.07*	102 ± 12
267	7x29	S	L	JNJ	41		83 ± 8	6.87 ± 0.14	65 ± 1*	6.48 ± 0.14	47 ± 5*	6.28 ± 0.23*	55 ± 5*	6.87 ± 0.19	63 ± 6*	6.42 ± 0.23*	82 ± 21	6.34 ± 0.12*	70 ± 8*
275	7x42	L	M	JNJ	41	1a	112 ± 10	7.68 ± 0.06*	76 ± 6*	6.91 ± 0.12	59 ± 12*	7.02 ± 0.06*	88 ± 2*	7.27 ± 0.27	56 ± 2*	7.34 ± 0.28*	50 ± 16	6.97 ± 0.21	65 ± 15*

Table S4. Mutations of negative charged wall residues. See Table S1 for more information.

GPR 139 res. pos.	GPCRdb generic residue number	WT AA	Mut AA	Surface Expression	$G\alpha_q$ activation						$G\alpha_{i1}$ activation					
					JNJ-63533054		TAK-041		Cmp1a		JNJ-63533054		TAK-041		Cmp1a	
					pEC_{50}	E_{max}	pEC_{50}	E_{max}	pEC_{50}	E_{max}	pEC_{50}	E_{max}	pEC_{50}	E_{max}	pEC_{50}	E_{max}
84	2x61	D	A	30 ± 7	7.42 ± 0.12	35 ± 8	6.99 ± 0.17	50 ± 12	7.04 ± 0.11	52 ± 10	6.84 ± 0.23	55 ± 10	6.99 ± 0.08	77 ± 21	6.81 ± 0.10	72 ± 11
84<br>+88<br>+89<br>+101<br>+105<br>+108<br>+268	2x61<br>+2x65<br>+2x66<br>+3x25<br>+3x29<br>+3x32<br>+7x35	D<br>+E<br>+D<br>+D<br>+E<br>+E<br>+E	A<br>+A<br>+A<br>+A<br>+A<br>+A<br>+A	31 ± 7	No signal	-0,2 ± 2					No signal	6 ± 4				
88	2x65	E	A	80 ± 26	7.14 ± 0.09	87 ± 7					7.11 ± 0.23	75 ± 3				
89	2x66	D	A	50 ± 9	7.35 ± 0.16	68 ± 3					7.40 ± 0.19	94 ± 12				
101	3x25	D	A	150 ± 5	7.47 ± 0.17	137 ± 8					7.37 ± 0.26	104 ± 7				
105	3x29	E	A	118 ± 7	7.35 ± 0.15	106 ± 5	7.07 ± 0.07	129 ± 2	6.91 ± 0.09	127 ± 9	7.44 ± 0.14	113 ± 11	7.17 ± 0.05	143 ± 25	7.40 ± 0.07	139 ± 12
108	3x32	E	A	89 ± 20	7.19 ± 0.14	83 ± 8	6.75 ± 0.07	121 ± 8	7.04 ± 0.03	120 ± 16	7.54 ± 0.28	101 ± 9	7.22 ± 0.22	139 ± 16	6.99 ± 0.18	102 ± 7
268	7x35	D	A	80 ± 6	6.23 ± 0.19	72 ± 4					6.34 ± 0.17	54 ± 6				

Table S5. Cryo-EM data collection, model refinement, and validation statistics.

	GPR139-miniG$_{s/q}$	GPR139-G$_i$	GPR139-miniG$_{s/q}$-GTP	GPR139-miniG$_{s/q}$-GDP
Data collection and processing				
Magnification	130,000	130,000	105,000	105,000
Voltage (kv)	300	300	300	300
Electron exposure (e-/Å^2)	60	60	60	60
Defocus range(μm)	-1.0 ~ -2.0	-1.0 ~ -2.0	-1.2 ~ -2.0	-1.0 ~ -2.0
Pixel size (Å)	0.52	0.52	0.416	0.416
Symmetry imposed	C1	C1	C1	C1
Final particles	110,337	250,483	67,676	129,015
Map resolution	3.2	3.2	3.3	3.8
FSC threshold	0.143	0.143	0.143	0.143
Refinement				
Initial model used (PDB code)	5DHG, 6KPF, 6UP7	5DHG, 6KPF		
Map sharping B factor (Å)	57.5	72.5	73.8	129.3
Model composition				
Non-hydrogen atoms	8,280	8,776	8287	8283
Protein residues	1,042	1,127	1034	1034
Ligand	JNJ-63533054	JNJ-63533054	JNJ-63533054	JNJ-63533054
B-factors				
Protein	105.3	104.1	69.36	120.56
Ligand	89.31	73.48	70.53	116.16
R.M.S. deviations				
Bond lengths(Å)	0.003	0.003	0.002	0.004
Bond angles (°)	0.596	0.568	0.557	0.664
Validation				
MolProbity score	2.05	1.99	1.84	2.17
Clash score	13.55	11.49	10.8	18.57
Poor rotamers (%)	0.11	0	0.56	0.67
Ramachandran plot				
Favored (%)	93.79	93.78	95.79	93.93
Allowed (%)	6.21	6.22	4.11	5.97

	GPR139-miniG$_{s/q}$	GPR139-G$_i$	GPR139-miniG$_{s/q}$-GTP	GPR139-miniG$_{s/q}$-GDP
Disallowed (%)	0	0	0.10	0.10
EMD code	EMD-32128	EMD-32127	EMD-32129	EMD-32130
PDB code	7VUH	7VUG	7VUI	7VUJ

References

1 Nehme, R. *et al.* Mini-G proteins: Novel tools for studying GPCRs in their active conformation. *PLoS One* **12**, e0175642, doi:10.1371/journal.pone.0175642 (2017).

2 Rasmussen, S. G. *et al.* Crystal structure of the beta2 adrenergic receptor-Gs protein complex. *Nature* **477**, 549-555, doi:10.1038/nature10361 (2011).

3 Maeda, S. *et al.* Development of an antibody fragment that stabilizes GPCR/G-protein complexes. *Nat Commun* **9**, 3712, doi:10.1038/s41467-018-06002-w (2018).

4 Mastronarde, D. N. Automated electron microscope tomography using robust prediction of specimen movements. *J Struct Biol* **152**, 36-51, doi:10.1016/j.jsb.2005.07.007 (2005).

5 Punjani, A., Rubinstein, J. L., Fleet, D. J. & Brubaker, M. A. cryoSPARC: algorithms for rapid unsupervised cryo-EM structure determination. *Nat Methods* **14**, 290-296, doi:10.1038/nmeth.4169 (2017).

6 Sanchez-Garcia, R. *et al.* DeepEMhancer: a deep learning solution for cryo-EM volume post-processing. *bioRxiv preprint*, doi:10.1101/2020.06.12.148296 (2020).

7 Lin, X. *et al.* Structural basis of ligand recognition and self-activation of orphan GPR52. *Nature* **579**, 152-157, doi:10.1038/s41586-020-2019-0 (2020).

8 Hua, T. *et al.* Activation and Signaling Mechanism Revealed by Cannabinoid Receptor-Gi Complex Structures. *Cell* **180**, 655-665 e618, doi:10.1016/j.cell.2020.01.008 (2020).

9 Miller, R. L. *et al.* The Importance of Ligand-Receptor Conformational Pairs in Stabilization: Spotlight on the N/OFQ G Protein-Coupled Receptor. *Structure (London, England : 1993)* **23**, 2291-2299, doi:10.1016/j.str.2015.07.024 (2015).

10 Pettersen, E. F. *et al.* UCSF Chimera--a visualization system for exploratory research and analysis. *J Comput Chem* **25**, 1605-1612, doi:10.1002/jcc.20084 (2004).

11 Emsley, P., Lohkamp, B., Scott, W. G. & Cowtan, K. Features and development of Coot. *Acta Crystallogr D Biol Crystallogr* **66**, 486-501, doi:10.1107/S0907444910007493 (2010).

12 Chen, V. B. *et al.* MolProbity: all-atom structure validation for macromolecular crystallography. *Acta Crystallogr D Biol Crystallogr* **66**, 12-21, doi:10.1107/S0907444909042073 (2010).

13 Nohr, A. C. *et al.* The GPR139 reference agonists 1a and 7c, and tryptophan and phenylalanine share a common binding site. *Scientific reports* **7**, 1128, doi:10.1038/s41598-017-01049-z (2017).

14 Masuho, I. *et al.* Distinct profiles of functional discrimination among G proteins determine the actions of G protein-coupled receptors. *Science signaling* **8**, ra123, doi:10.1126/scisignal.aab4068 (2015).

15 Friesner, R. A. *et al.* Glide: A New Approach for Rapid, Accurate Docking and Scoring. 1. Method and Assessment of Docking Accuracy. *Journal of Medicinal Chemistry* **47**, 1739-1749, doi:10.1021/jm0306430 (2004).

16 Friesner, R. A. *et al.* Extra Precision Glide: Docking and Scoring Incorporating a Model of Hydrophobic Enclosure for Protein−Ligand Complexes. *Journal of Medicinal Chemistry* **49**, 6177-6196, doi:10.1021/jm051256o (2006).

17 Halgren, T. A. *et al.* Glide: A New Approach for Rapid, Accurate Docking and Scoring. 2. Enrichment Factors in Database Screening. *Journal of Medicinal Chemistry* **47**, 1750-1759, doi:10.1021/jm030644s (2004).

18 *Schrödinger Release 2020-2: LigPrep.* (Schrödinger, LLC, 2020).

19 *Schrödinger Release 2020-2: Glide.* (Schrödinger, LLC, 2020).

20 Jo, S., Kim, T., Iyer, V. G. & Im, W. CHARMM-GUI: a web-based graphical user interface for CHARMM. *J Comput Chem* **29**, 1859-1865, doi:10.1002/jcc.20945 (2008).
21 Wu, E. L. *et al.* CHARMM-GUI Membrane Builder toward realistic biological membrane simulations. *J Comput Chem* **35**, 1997-2004, doi:10.1002/jcc.23702 (2014).
22 Maier, J. A. *et al.* ff14SB: Improving the Accuracy of Protein Side Chain and Backbone Parameters from ff99SB. *J Chem Theory Comput* **11**, 3696-3713, doi:10.1021/acs.jctc.5b00255 (2015).
23 Wang, J., Wolf, R. M., Caldwell, J. W., Kollman, P. A. & Case, D. A. Development and Testing of a General Amber Force Field. *J Comput Chem* **25**, 1157-1174 (2004).
24 Goddard, T. D. *et al.* UCSF ChimeraX: Meeting modern challenges in visualization and analysis. *Protein Sci* **27**, 14-25, doi:10.1002/pro.3235 (2018).
25 Kim, K. *et al.* Structure of a Hallucinogen-Activated Gq-Coupled 5-HT2A Serotonin Receptor. *Cell* **182**, 1574-1588 e1519, doi:10.1016/j.cell.2020.08.024 (2020).
26 Xia, R. *et al.* Cryo-EM structure of the human histamine H1 receptor/Gq complex. *Nat Commun* **12**, 2086, doi:10.1038/s41467-021-22427-2 (2021).
27 Maeda, S., Qu, Q., Robertson, M. J., Skiniotis, G. & Kobilka, B. K. Structures of the M1 and M2 muscarinic acetylcholine receptor/G-protein complexes. *Science* **364**, 552-557, doi:10.1126/science.aaw5188 (2019).
